# Reactions of cobalt(ii) chloride and cobalt(ii) acetate with hemisalen-type ligands: ligand transformation, oxidation of cobalt and complex formation. Preliminary study on the cytotoxicity of Co(ii) and Co(iii) hemisalen complexes†

**DOI:** 10.1039/d2ra07089h

**Published:** 2023-03-16

**Authors:** Magdalena Siedzielnik, Monika Pawłowska, Mateusz Daśko, Hubert Kleinschmidt, Anna Dołęga

**Affiliations:** a Department of Inorganic Chemistry, Chemical Faculty, Gdansk University of Technology Narutowicza 11/12 80-233 Gdansk Poland magdalena.siedzielnik@pg.edu.pl; b Department of Pharmaceutical Technology and Biochemistry, Chemical Faculty, Gdansk University of Technology Narutowicza 11/12 80-233 Gdansk Poland

## Abstract

Several molecular cobalt(ii) complexes, one Co(ii) coordination polymer and one ionic cobalt(iii) complex with imine hemisalen ligands were synthesized. The hemisalen ligands were synthesized from *o*-vanillin (*o*VP) and diverse aminopyridines (compounds HL1–HL4) or aminophenol (compound HL5). It was observed that cobalt(ii) chloride in dry acetonitrile catalyzes a transformation of HL1 and HL3 instead of complex formation. The conversion of these imines proceeded *via* self-cyclization to *N*-2′′-pyridyl-2,6-dioxo-9-aza-[*c*,*g*]di-2′-methoxybenzo nonan or its methyl derivative as the major product. The remaining reactions were performed using imines HL1–HL5 and cobalt(ii) acetate Co(Ac)_2_ in methanol or DMSO/acetonitrile resulting in forming a series of cobalt complexes. The following series of compounds was obtained: two similar tetrahedral molecular Co(ii) complexes [Co(L1)_2_] and [Co(L3)_2_], one trinuclear, mixed-ligand Co_3_(Ac)_2_(L4)_2_(*o*VP)_2_, one coordination polymer {Co(L2)_2_}_∞_ and finally one octahedral anionic Co(iii) complex [HNEt_3_][Co(L5)_3_]. The latter complex formed in a cobalt(ii) acetate reaction with a hemisalen HL5 derived from *o*VP and 2-aminophenol. The molecular structures of all compounds were confirmed by X-ray diffraction, and the cytotoxicity of Co(ii) and Co(iii) complexes towards cancer cell lines HCT116, HL-60 and normal cell line MRC-5 was studied.

## Abstract 2

Dependent on the cobalt precursor, hemisalen complexes tend to transform into polycyclic compounds or bind to the metal ion to form coordination complexes of diverse nuclearity.

## Graphical abstract

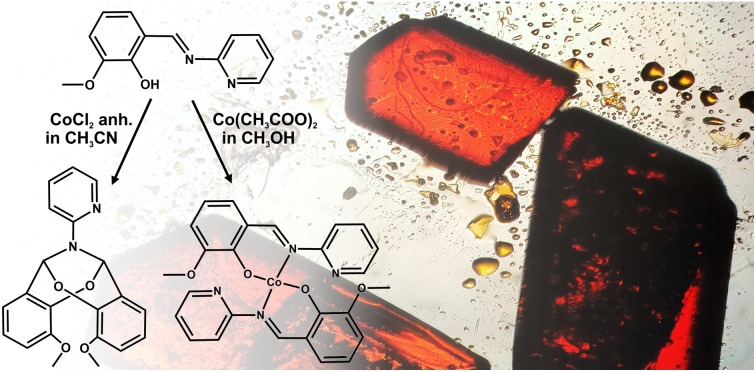

## Introduction

Cobalt(ii) and cobalt(iii) complexes with Schiff bases have gained much attention as promising catalysts for the synthesis of a variety of organic compounds,^1,2^ polymerization, *e.g.* of ethylene,^3^ proton reduction,^4,5^ alkylation reactions *etc.*^4^ Moreover, these compounds show antibacterial and anticancer properties.^6–8^

**Scheme 1:**
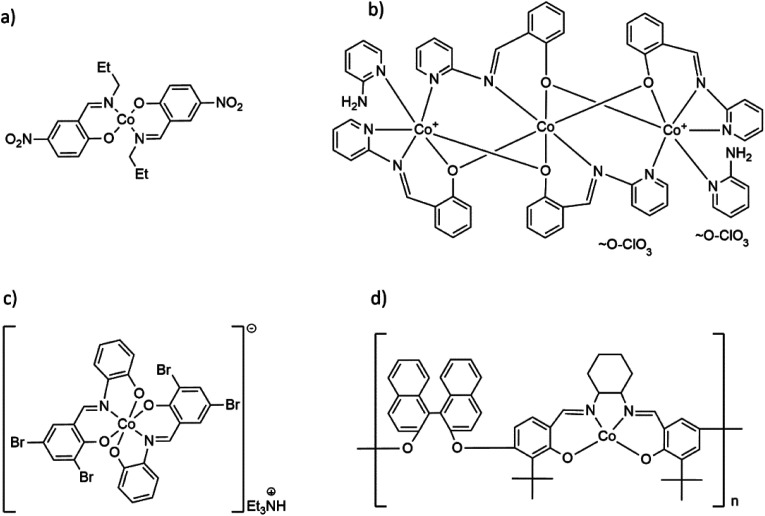
Formulas of selected Co(ii) and Co(iii) complexes with hemisalen and salen-type ligands: (a) molecular Co(ii) complex Co(C_10_H_11_N_2_O_3_)_2_,^17^ (b) trinuclear [Co_3_(C_13_H_9_N_2_)_4_(2-AP)_2_](ClO_4_)_2_,^18^ (c) cationic, mononuclear [Co(C_13_H_7_Br_2_N)_2_](Et_3_NH) complex^19^ and (d) Co(ii) coordination polymer with salen-type ligand [CoC_48_H_44_O_4_N_2_]_*n*_.^20^

Schiff base complexes with transition metals are of great interest not only for their coordination chemistry and structural diversity but also as functional compounds with a wide range of applications.^9–11^ Potential use as catalysts or biologically active compounds has contributed to the synthesis of Co(ii) and Co(iii) complexes with Schiff bases from simple mononuclear complexes to coordination polymers with different dimensionality (Scheme 1).^12–17^

Schiff bases feature exciting properties related to the simultaneous presence of proton-donor and proton-acceptor groups, the possibility of formation of inter- and intramolecular hydrogen bonds and participation in proton transfer processes.^16–24^ Based on these properties, imines are widely used in organic processes, such as the addition of organometallic reagents to C=N bond,^16,20,21,25,26^ hydrolysis to aldehyde/ketone and amine,^21,27,28^ self-condensation,^25,29–32^ complexation reactions with both main groups and transition metals,^21,33,34^ pH- and metal ion-dependent hydrolysis on the metal center during the formation of the coordination complex.^35–38^

In our previous work, we described the immediate hydrolysis of the imine HL1 (2-methoxy-6-(*E*-2-pyridyliminomethyl)-phenol) in the presence of nickel cations. This resulted in the formation of various heteroligand complexes.^39^ Since the results were unexpected to us, we decided to study the behavior of cobalt(ii) salts towards similar imines. In this work we describe the reactions that undergo between cobalt chloride or cobalt acetate and selected hemi-salen-type imines in various solvents; we indicate important differences in the reaction course that result from a given choice of reagents and solvents. During our studies, we have noticed that diphenolate ligands stabilize the +3 oxidation state of cobalt. We have therefore decided to take the advantage of the possibility to isolate similar Co(ii) and Co(iii) coordination compounds and compare the cytotoxic effects of labile Co(ii) and inert Co(iii) complexes towards abnormal, cancer HCT116, HL-60, and normal MRC-5 cell lines.

## Results and discussion

### Synthesis

**Scheme 2:**
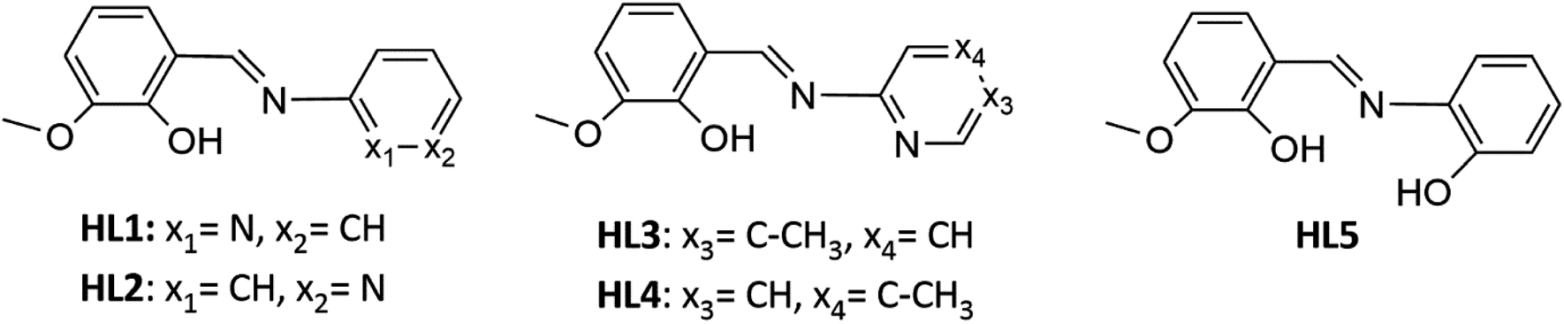
Formulas of the ligands HL1–HL5.

The reactivity of selected Schiff bases toward cobalt(ii) salts was studied. The syntheses were carried out using five imines, all of which are the derivatives of *o*-vanillin. The formulas of the compounds are shown in Scheme 2.

Initially, we performed the reaction between cobalt(ii) chloride and HL1 in anhydrous acetonitrile. As described in the experimental section, the reaction mixture was clear and emerald green. The first product we obtained was a colorless bicyclic C1A that resulted from the cyclization of HL1. TLC was used to monitor the progress of the reaction. The formation of the cyclic compound C1A was observed immediately upon the addition of the cobalt salt. The crystalline product formed directly in the reaction mixture at a low temperature (+4 °C). So far, there has been no literature information that the cobalt salts facilitate the transformation of Schiff bases into cyclic compounds, as indicated by our studies. In 1998 Filarowski and co-workers revealed that after a few months at room temperature, Schiff base (from salicylaldehyde and methylamine) undergoes self-condensation to the *N*-methylo-2,6-dioxo-9-aza-[*c*,*g*]dibenzo^1,3,3^ nonan. This cyclic condensation, probably with simultaneous elimination of one molecule of methylamine, proceeds in mild conditions.^29^ It is worth pointing out that only few compounds of this type have been described in the literature and their method of synthesis is mainly based on the addition of a suitable aldehyde to ammonium acetate; in some cases before the addition of the aldehyde, an ester was added to functionalize the nitrogen atom. Such reactions were catalyzed either with β-naphthol and a benzoquinone derivative,^40^ with concentrated sulfuric(VI) acid,^41^ or proceeded without the addition of a catalyst at the increased temperature (boiling ethanol).^31^ Another example of acid-catalyzed cyclization has been published very recently.^32^ Concerning the results described in the literature, we tested whether the dissolution of the HL1 ligand in acetonitrile would lead to the non-catalyzed self-cyclization in a time-frame reasonable for a chemist. During two years, we have not observed the formation of the cyclic compound. Obviously, in the case of the self-cyclization reaction of HL1, cobalt chloride served as a catalyst.

Interestingly, the imine derivative of thiovanillin seems more prone to similar reaction of condensation since a cyclic compound very similar to C1A was obtained with a good yield in a non-catalyzed reaction between a thiovanillin and 3-AP conducted for one hour in dry, boiling ethanol.^31^

**Scheme 3:**
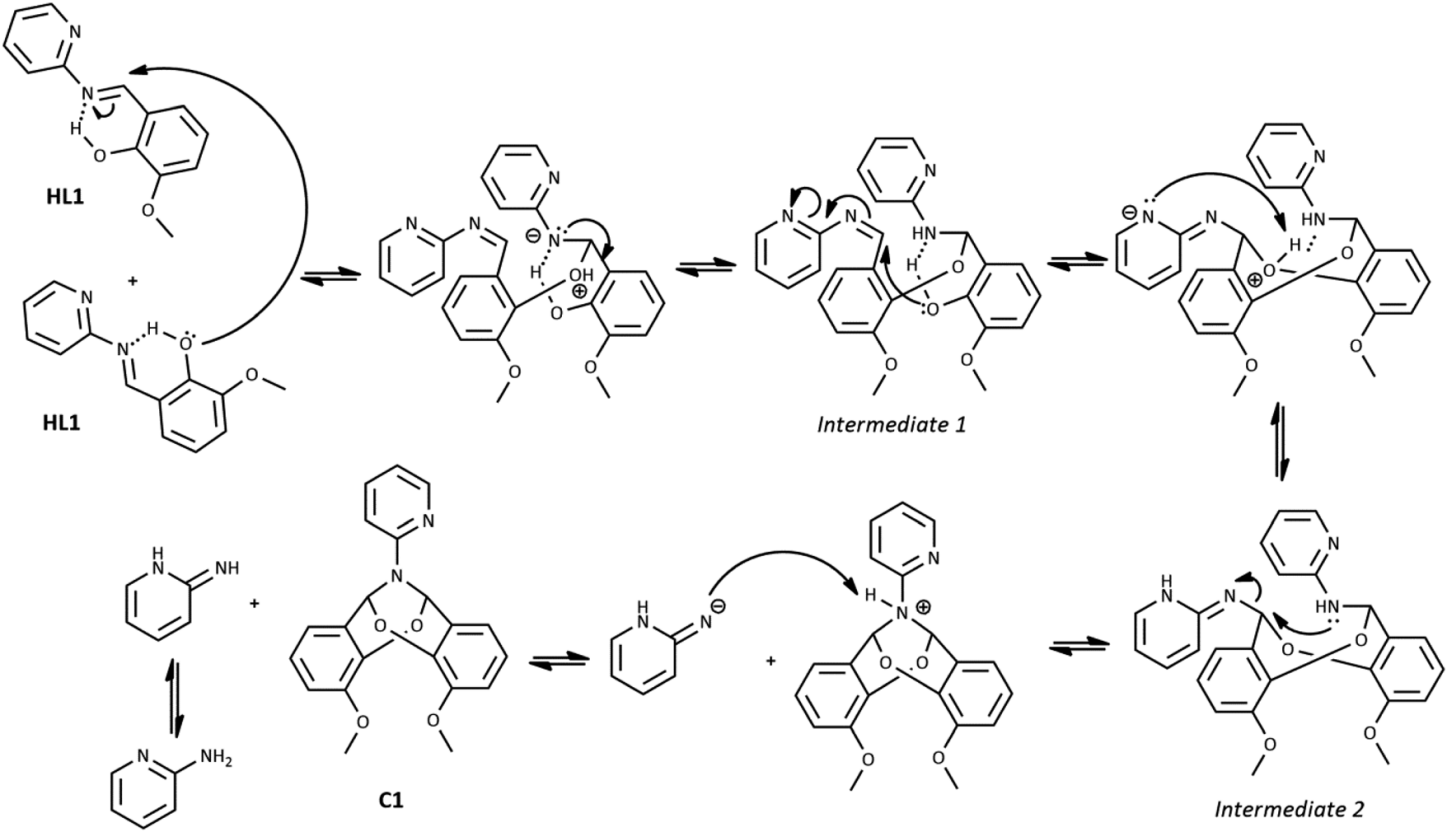
The probable mechanism of cyclization of imine HL1.

The possible mechanism of the self-cyclization reaction is presented in Scheme 3. Considering the probable formation of an intramolecular hydrogen bond between the imine bond N atom and hydroxyl group of HL1, the electrophilicity of imine carbon is increased. Therefore, the nucleophilic attack of the hydroxyl group of the second HL1 molecule is possible, leading to the generation of Intermediate 1. Due to the presence of the second imine group within Intermediate 1, the subsequent intramolecular nucleophilic attack of free hydroxyl group and formation of Intermediate 2 is possible. Finally, the nucleophilic attack of the N atom on the CH group may occur, leading to the elimination of the 2-aminopyridine and the formation of the bicyclic compound C1A. Another likely mechanism of C1A formation, which includes the reaction between HL1 and *o*-vanillin generated as a result of partial hydrolysis of HL1, is illustrated in Scheme 1S in ESI† (as in ref. 31).

**Scheme 4:**
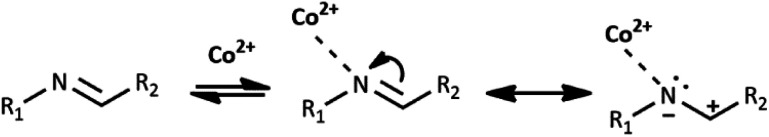
Influence of Co(ii) ions on cyclization of imine HL1.

Co(ii) ions may further facilitate cyclization by direct coordination with imine nitrogen. The complexation should lower the electron density within the imine bond and increase the electrophilicity of the imine carbon, as shown in Scheme 4. We tentatively suggest the mechanism of catalysis with Co(ii) ions in Scheme 2S.† Our further experimental efforts support this mechanism. We observed the cyclization not only for HL1 but also for HL3 and the same reaction conditions. We describe the cyclization product C1B obtained from HL3 in the Experimental and ESI (Table 1S and Fig. 2S†). Moreover, cyclization was not observed for HL2 – perhaps because the position of pyridyl N2 in HL2 does not allow the formation of the chelating Co(ii) complex indicated in Scheme 1S.†

From the reaction mixture of cobalt chloride with HL1 in acetonitrile, except for C1A, we have also isolated the known cobalt(ii) complex C2 [Co(2-AP)Cl_3_]^−^[2-APH]^+^, which explains the “fate” of the 2-aminopyridine resulting from the reaction illustrated in Scheme 3.^42^ We have also observed a certain amount of green, amorphous precipitate in the reaction of HL3 with cobalt(ii) chloride.

Other reactions of imine compounds may undergo within the studied system, however we did not observe them. The example is a nucleophilic addition of the amine to the –C=N– bond with the formation of aminal, as proved for similar imines.^43^

**Scheme 5:**
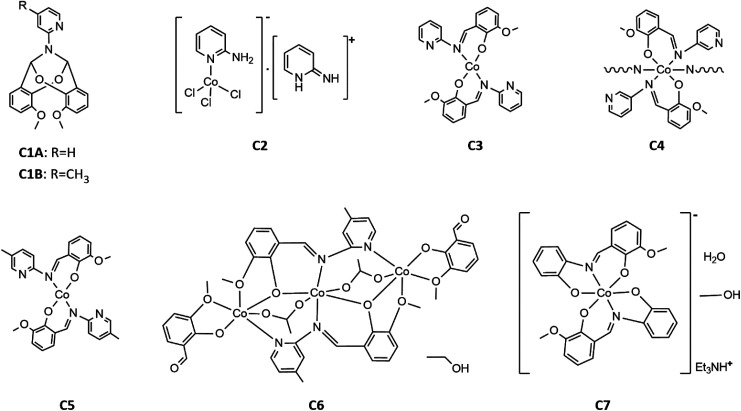
Formulas of the obtained compounds C1A, C1B and complexes C2–C7.

Finally, to produce the desired Co(ii) imine complexes, we decided to change the Co(ii) salts and solvents. The cobalt(ii) complexes C3–C7 formed in simple reactions between: Co(CH_3_COO)_2_·4H_2_O and imines HL1-HL5 deprotonated by triethylamine in methanol or ethanol. Four complexes: C3, C5, C6 and C7 were isolated by crystallization at low temperature (+4 °C). Complex C4 crystallized at once at RT (room temperature) as the fine crystalline powder. Compounds C3, C5, C6, and C7, were characterized by X-ray diffraction analysis (see Section crystal structures and Hirshfeld surfaces). In the case of compound C4, yellow powder was insoluble in the tested organic and inorganic solvents such as DMSO, DMF, MeOH, EtOH, acetone, THF, DCM, H_2_O, *etc*. Based on infrared spectra, elemental analysis, our previous results for Cu(ii) complexes,^34^ and the insolubility of C4, we suggest that the reaction between deprotonated HL2 and cobalt(ii) acetate yielded a coordination polymer. The formulas of the complexes are displayed in Scheme 5.

The reaction between HL3 and cobalt acetate in methanol leads to the formation of Co(ii) complex C5. Due to the low yield, we decided to replace methanol with a mixture of DMSO and acetonitrile. This modification increased the output of the synthesis from 10% to 45%.

It is important to note that the reaction of cobalt(ii) acetate with imine HL5 derived from *o*-vanillin and 2-aminophenol leads to the cobalt(iii) complex. We did not apply any oxidizing reagents during the synthesis, nevertheless the oxidation proceeded quickly. Though not emphasized, the change of the cobalt oxidation state was observed previously for the reactions of other diphenol imines with Co(ii) salts.^16,19^ Therefore, we suggest that diphenolate ligands strongly stabilize the +3 oxidation number of cobalt.

### Crystal structures and Hirshfeld surfaces

**Fig. 1:**
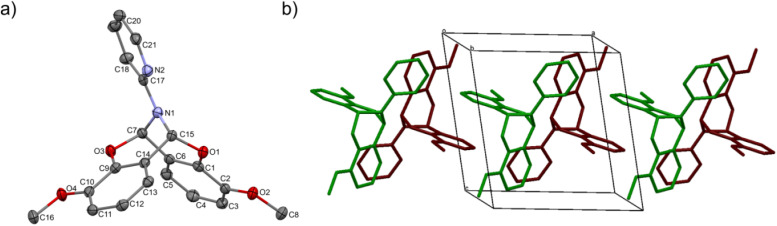
Crystal structure of C1A: (a) molecular structure of C1A with the numbering scheme; important bond lengths [Å]: O3–C9 1.3704(13), O3–C7 1.4558(12), O1–C1 1.3694(13), O1–C15 1.4433(12), N1–C17 1.4058(13); important angles [°]: C1–O1–C15 112.85(8), C17–N1–C7 121.05(9), C17–N1–C15 119.57(9), C7–N1–C15 109.25(8); (b) crystal packing. Hydrogen atoms were omitted for clarity.

**Fig. 2:**
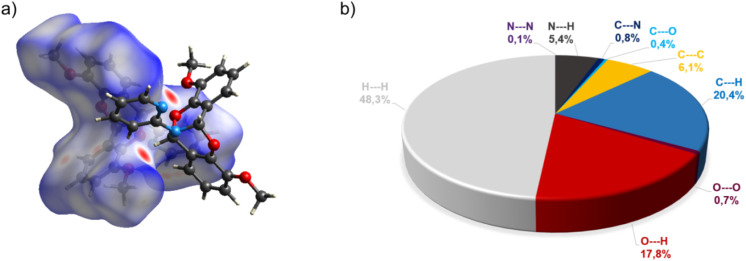
(a) Hirshfeld surfaces of C1A. Red color: normalized contact distances *d*_norm_ shorter than the sum of van der Waals radii (*d*_norm_ = −0.230), white color: van der Waals contacts (*d*_norm_ = 0.441), and blue color: normalized contact distances exceeding the sum of van der Waals radii (*d*_norm_ = 1.156); (b) Hirshfeld surface fingerprint decompositions showing the main types of interactions for C1A.

The crystal structure of bicyclic compound C1A is presented in Fig. 1. Compound C1A crystallizes as well-formed, colorless crystals from an anhydrous acetonitrile solution of HL1 and CoCl_2_. C1A crystallizes in a triclinic system. The asymmetric unit contains one molecule. The compound consists of six connected rings, including two benzene rings, one pyrimidine ring, two six-membered heterocyclic rings, and one eight-membered heterocyclic ring. The rings are either fused or bridged. The geometry around the nitrogen atom is flat, and the sum of the corresponding angles is approximately 360°, which demonstrates the sp^2^ hybridization of the N1 atom. The analysis of the Hirshfeld surface presented in Fig. 2 indicates that the principal interactions between molecules in compound C1A are van der Waals forces. The decomposed fingerprint plot shows that the hydrophobic H⋯H (48.3%) interactions are dominating in the crystal packing with C⋯H (20.4%) interactions representing the next highest contribution. Red areas on the Hirshfeld surface correspond to more directional interactions C7–H7⋯O4. The similar molecular structure of C1B is illustrated in Fig. 1S (ESI†).

The crystal structure of C2 was described elsewhere.^42^ We included the low-temperature crystal data in ESI as Table 1S.† The relevant bond lengths and angles are presented in Fig. 2S (ESI†).

**Fig. 3:**
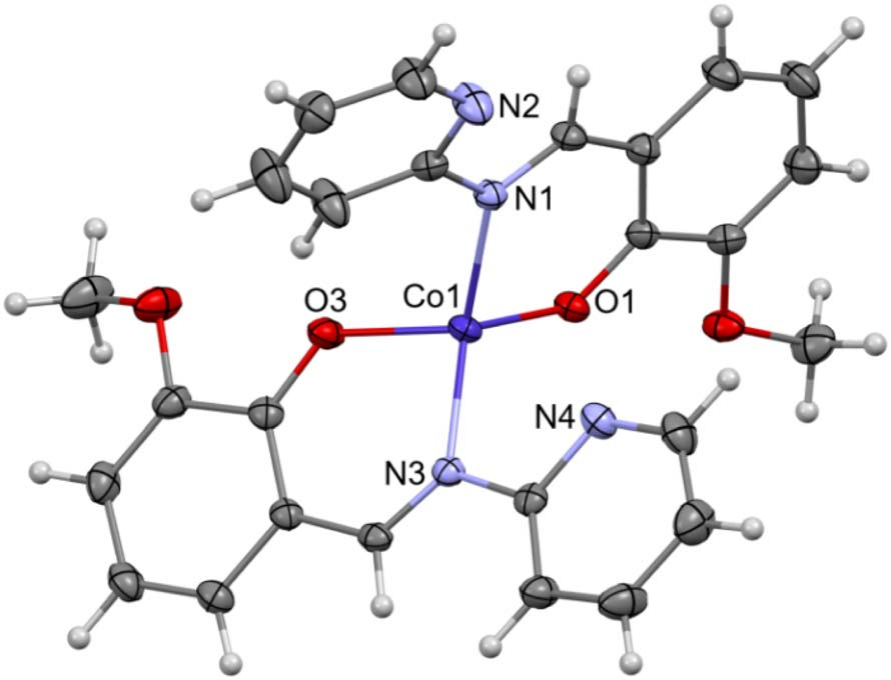
Molecular structure of C3 with the numbering scheme; important bond lengths [Å]: Co1–O1 1.9255(10), Co1–O3 1.9389(10), Co1–N3 1.9990(12), Co1–N1 2.0108(13); important angles [°]: O1–Co1–O3 113.81(5), O1–Co1–N3 118.03(5), O3–Co1–N3 91.08(5), O1–Co1–N1 95.09(4), O3–Co1–N1 111.24(5), N3–Co1–N1 128.54(5).

The crystal structure of C3 is shown in the Fig. 3. Well-formed, red, monoclinic crystals were grown from methanol. Asymmetric unit contains one molecule. Compound C3 is an example of a mononuclear cobalt(ii) complex, where two imine ligands (L1) coordinate to the cobalt atom in a distorted tetrahedral geometry. In our previous paper about polynuclear Ni(ii)–L1 complexes we anticipated the potential distortion of C3. Previous DFT calculations for a series of transition metal complexes with L1 indicated quasi-three-coordination of one of the imine ligands.^39^ Now, we are able to present the experimental crystal structure, which confirms that the distortion results from the interaction between Co(ii) and one of the pyridyl nitrogens (N4). Experimental Co(ii)⋯N4 distance equals to 2.711(1) Å. The previous DFT calculations “foresaw” the coordination geometry of Co(ii) in C3 very well.^39^ The detailed comparison between experimental and theoretical structure is included in ESI as Fig. 3S and Table 2S.†

**Fig. 4:**
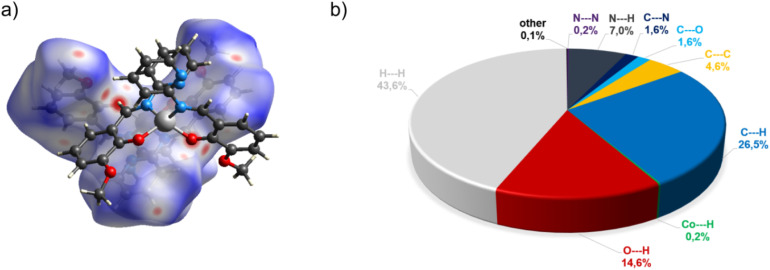
(a) Hirshfeld surfaces of C3. Red color: normalized contact distances *d*_norm_ shorter than the sum of van der Waals radii (*d*_norm_ = −0.199), white color: van der Waals contacts (*d*_norm_ = 0.454), and blue color: normalized contact distances exceeding the sum of van der Waals radii (*d*_norm_ = 1.341); (b) Hirshfeld surface fingerprint decompositions showing the main types of interactions for C3.

In the diagram presented in Fig. 4, we can see as many as ten spots that indicate short intermolecular contacts between the two molecules of C3. The most intense red areas on the Hirshfeld surface correspond to the interactions C21–H21⋯O1 and C21–H21⋯O2. The decomposed fingerprint plot shows that the hydrophobic H⋯H (43.6%) interactions are the crucial factor in the crystal packing, with C⋯H (26.5%) representing the next highest contribution. The Hirshfeld analysis suggests less typical Co⋯H contact between the molecules.

**Fig. 5:**
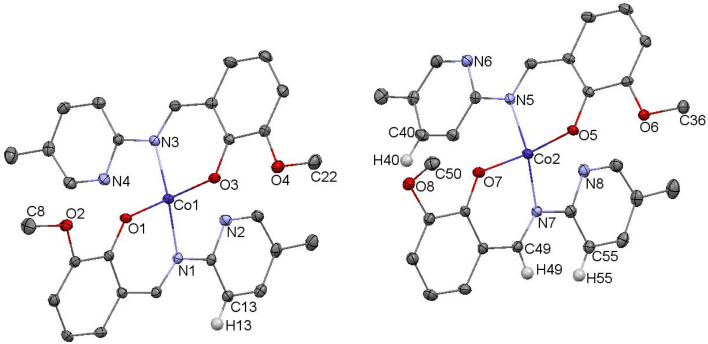
Molecular structure of C5 with the numbering scheme; important bond lengths [Å]: Co1–O1 1.939(2), Co1–O3 1.947(2), Co1–N3 1.988(2), Co1–N1 1.997(2), Co2–O5 1.910(2), Co2–O7 1.932(2), Co2–N7 1.985(2), Co2–N5 2.000(2); important angles [°]: O1–Co1–O3 102.71(9), O1–Co1–N3 111.40(9), O3–Co1–N3 93.39(9), O1–Co1–N1 92.55(9), O3–Co1–N1 113.90(9), N3–Co1–N1 138.98(10), O5–Co2–O7 113.81(9), O5–Co2–N7 117.36(10), O7–Co2–N7 93.04(9), O5–Co2–N5 97.16(9), O7–Co2–N5 106.28(9), N7–Co2–N5 129.27(10). Hydrogen atoms were omitted for clarity.

The crystal structure of complex C5 is presented in the Fig. 5. C5 crystallizes as tiny orange-red crystals. Similar to C3, C5 is an example of tetrahedral Co(ii) complex. In the independent part of the unit cell two conformers of C5 are found. The two molecules of C5 differ exclusively in the orientation of one of their methoxy groups (carbon atoms labelled C8/C50). The observed rotation allows the formation of additional C–H⋯O contacts between the neighboring molecules.

**Fig. 6:**
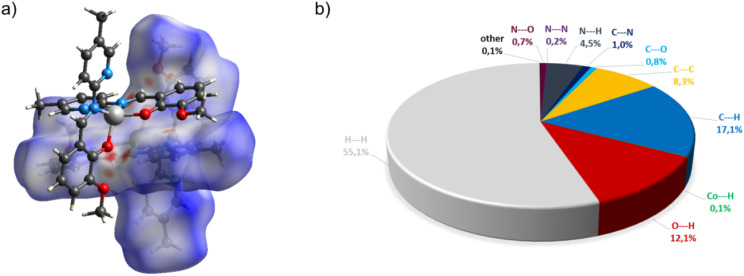
(a) Hirshfeld surfaces of C5. Red color: normalized contact distances *d*_norm_ shorter than the sum of van der Waals radii (*d*_norm_ = −0.212), white color: van der Waals contacts (*d*_norm_ = 0.470), and blue color: normalized contact distances exceeding the sum of van der Waals radii (*d*_norm_ = 1.317). (b) Hirshfeld surface fingerprint decompositions showing the main types of interactions for C5.

In the diagram presented in Fig. 6, we see the red spots that indicate short intermolecular contacts between the cobalt(ii) complex C5 molecule and its neighboring molecule. The most intense red areas on the Hirshfeld surface correspond to the interactions between C13–H13⋯O5, C55–H55⋯O3, C49–H49⋯O3, and C40–H49⋯O4. The decomposed fingerprint plot shows that the hydrophobic H⋯H (55.1%) interactions are again dominating in the crystal packing, with C⋯H (17.1%) interactions representing the next highest contribution.

**Fig. 7:**
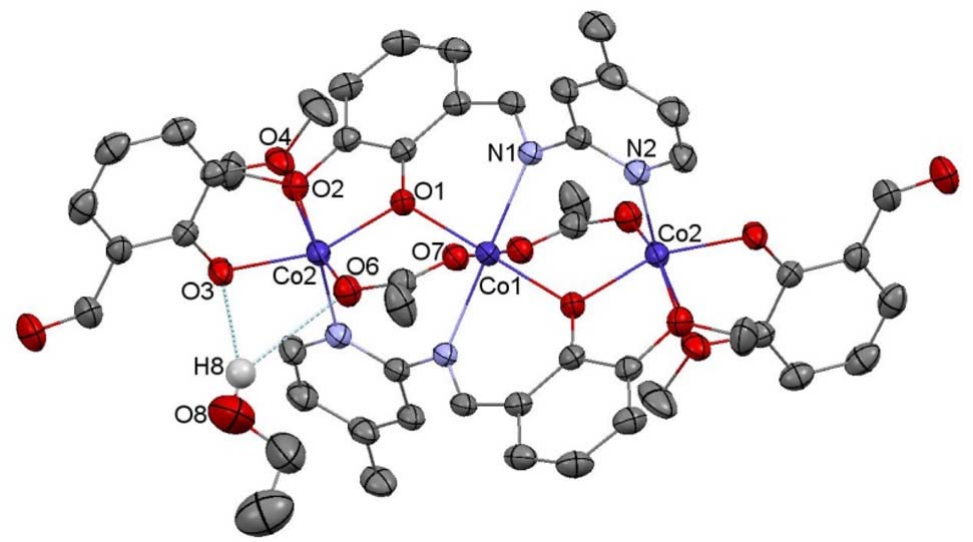
Molecular structure of C6 with the partial numbering scheme; hydrogen atoms were omitted for clarity; important bond lengths [Å]: Co1–O1^*i*^ 2.015(2), Co1–O1 2.015(2), Co1–O7 2.083(3), Co1–O7^*i*^ 2.083(3), Co1–N1^*i*^ 2.174(3), Co1–N1 2.174(3), Co2–O3 1.962(3), Co2–O1 1.998(2), Co2–O6 2.004(3), Co2–N2^*i*^ 2.094(3), Co2–O4 2.226(3); important angles [°]: O1^*i*^–Co1–O1 180, O1^*i*^–Co1–O7 90.00(10), O1–Co1–O7 90.00(10), O1^*i*^–Co1–O7^*i*^ 90.00(10), O1–Co1–O7^*i*^ 90.00(10), O7–Co1–O7^*i*^ 180, O1^*i*^–Co1–N1^*i*^ 84.18(10), O1–Co1–N1^*i*^ 95.82(10), O7–Co1–N1^*i*^ 89.29(11), O7^*i*^–Co1–N1^*i*^ 90.71(11), O1^*i*^–Co1–N1 95.82(10), O1–Co1–N1 84.48(10), O7–Co1–N1 90.71(11), N1^*i*^–Co1–N1 180.00(9), O3–Co2–O1 157.90(12), O3–Co2–O6 98.16(13), O1–Co2–O6 99.62(12), O3–Co2–N2^*i*^ 94.84(12), O1–Co2–N2^*i*^ 94.39(12), O6–Co2–N2^*i*^ 101.39(13), O3–Co2–O4 77.42(10), O1–Co2–O4 90.03(10), O6–Co2–O4 88.47(12), N2^*i*^–Co2–O4 168.30(12). *i*: 1−*x*, 1−*y*, 1−*z*.

**Fig. 8:**
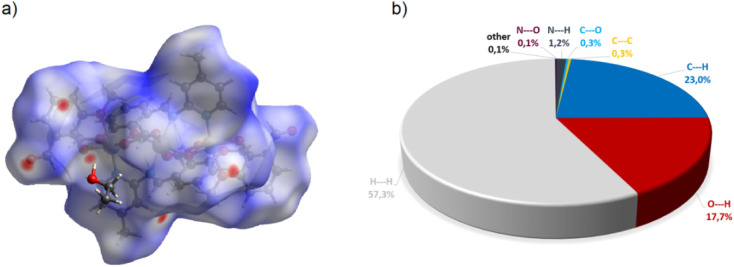
(a) Hirshfeld surfaces of C6. Red color: normalized contact distances *d*_norm_ shorter than the sum of van der Waals radii (*d*_norm_ = −0.186), white color: van der Waals contacts (*d*_norm_ = 0.573), and blue color: normalized contact distances exceeding the sum of van der Waals radii (*d*_norm_ = 1.767); (b) Hirshfeld surface fingerprint decompositions showing the main types of interactions for C6.

Compound C6, shown in Fig. 7, crystallizes in a monoclinic system. The unit cell contains half of the molecule. The trinuclear complex contains two anions of deprotonated imine (L4), two acetate ions, and two terminal *o*-vanillinate anions coordinated to the terminal Co(ii) in a chelating mode. C6 crystallizes as an ethanol solvate. Imine ligand bridges all three Co(ii) ions utilizing three different O/N donor atoms. The metal ions are also kept together by acetate anions, which results in a relatively short intermetallic separation of 3.332 Å, however these distances do not indicate metal–metal interactions. The central cobalt atom has a slightly distorted octahedral coordination geometry and is hexacoordinated by two imine nitrogen atoms, two phenolate oxygen atoms, and two acetate oxygen atoms. The coordination number of the terminal cobalt atoms is also six. In the diagram presented in Fig. 8, we illustrated short intermolecular contacts between the trinuclear cobalt(ii) complex C6 molecule and its neighboring molecules. The most intense red areas on the Hirshfeld surface correspond to classical hydrogen bonding interactions between O8–H8⋯O3, evidence of interaction between the molecular complex and the ethanol molecule.

**Fig. 9:**
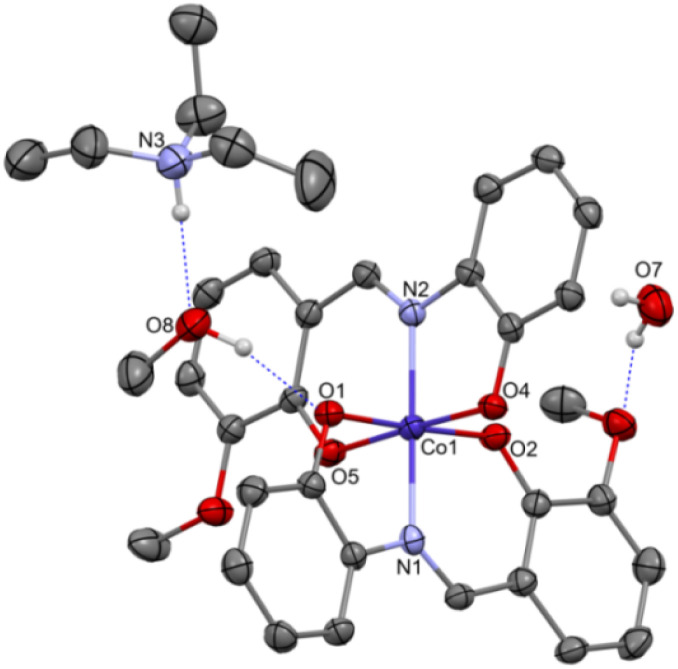
Molecular structure of C7 with the partial numbering scheme; hydrogen atoms were omitted for clarity; important bond lengths [Å]: Co1–O1 1.903(2), Co1–O2 1.892(2), Co1–O4 1.892(2), Co1–O5 1.894(2), Co1–N1 1.897(3), Co1–N2 1.897(3); important angles [°]: O2–Co1–O4 89.85(11), O2–Co1–O5 91.23(11), O4–Co1–O5 177.49(11), O2–Co1–N1 95.99(11), O4–Co1–N1 89.56(11), O5–Co1–N1 88.08(11), O2–Co1–N2 87.65(11), O4–Co1–N2 86.59(11), O5–Co1–N2 95.71(12), N1–Co1–N2 174.70(12), O2–Co1–O1 177.68(11), O4–Co1–O1 89.49(11), O5–Co1–O1 89.52(11), N1–Co1–O1 86.23(11), N2–Co1–O1 90.10(11).

**Fig. 10:**
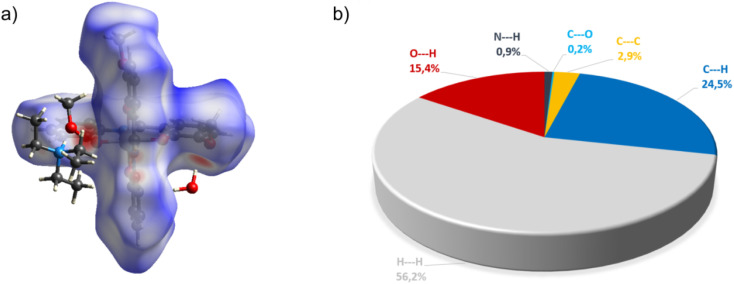
(a) Hirshfeld surfaces of C7. Red color: normalized contact distances *d*_norm_ shorter than the sum of van der Waals radii (*d*_norm_ = −0.719), white color: van der Waals contacts (*d*_norm_ = 0.468), and blue color: normalized contact distances exceeding the sum of van der Waals radii (*d*_norm_ = 1.396); (b) Hirshfeld surface fingerprint decompositions showing the main types of interactions for C7.

Complex C7 crystallizes in a monoclinic system. The unit cell contains four entities. The asymmetric unit is composed of a Co(iii) ion coordinated by the two imino ligands (L5) in an octahedral geometry. The charge of the complex anion is neutralized by the triethylammonium cation. There are also molecules of solvents: methanol and water within the crystal lattice (Fig. 9). In the diagram presented in Fig. 10, we illustrated short intermolecular contacts between the cobalt(iii) complex C7 molecule and its neighboring molecules. The most intense red areas on the Hirshfeld surface correspond to hydrogen bonds O8–H8⋯O1 and O7–H7A⋯O2 with the molecules of accompanying solvents. The relatively strong hydrogen bonding interactions are multiple and contribute significantly to the attractive forces. Such strong interactions between solvent, water molecules, and complex molecules have a stabilizing effect on the whole structure.

### Lipophilicity

Lipophilicity is one of the most studied physicochemical properties because it allows to predict chemical compound's fate in living organisms. Lipophilicity is a crucial property in drug design to obtain the optimal properties required to achieve a molecular target in cells.^44^

The lipophilicity of HL1, HL3, HL5, C3, C5, and C7 was calculated by the free access web tool SwissADME server (Swiss Institute of Bioinformatics, Lausanne, Switzerland). Moreover, SwissADME was used to predict drug-like physicochemical properties of these compounds based on Lipinski's Rule of Five (molecular weight <500, log *P* or coefficient partition between −5 and 5, H-bond donors <5, and H-bond acceptors <10).^45,46^ Compounds violating more than one of these rules may demonstrate problems with bioavailability.

Our calculations show that the selected compounds fulfill the desired drug-like physicochemical features. Although the complexes C3, C5, and C7 violate one of the requirements – their molar weights exceed 500 – they still satisfy Lipinski's Rule. There are several examples of clinical drugs of big molecular mass *e.g.*: vincristine, vinblastine (>800 g mol^−1^), irinotecan, and daunorubicin (>500 g mol^−1^).

Using the MLOGP module, the highest lipophilicity within ligands was demonstrated for compound HL3 (log *P* = 1.83), while the lowest lipophilicity in this group was calculated for HL1 (log *P* = 1.56). In the group of coordination complexes, the highest lipophilicity was obtained for complex C5 (log *P* = 2.83) and the lowest for C7 (log *P* = 1.74), following our expectations (C7 is ionic). log *P* of all presented cobalt complexes and their ligands is between 1.56 and 2.83, suggesting that they should enter the cell to reach their molecular target (Table 1).

**Table 1:** Calculated physicochemical properties of selected compounds based on Lipinski's rule of five

Compound	Molecular weight (g mol^−1^)	log *P* (MLOGP)	Number of H-bond donors	Number of H-bond acceptors
HL1	228.25	1.56	1	4
HL3	242.27	1.83	1	4
HL5	243.26	1.67	2	4
C3	513.41	2.43	0	6
C5	541.46	2.83	0	6
C7	541.42	1.74	0	6

### Cytotoxicity towards human cancer and normal cells

Neoplastic diseases are still a challenge for medicine and pharmacy. Despite many medications available in the clinic, there is still no drug that is effective in treating every type of cancer and is safe for the patient. Therefore, new drugs are constantly searched for, even if they can help a narrow group of patients. One of the oldest chemotherapeutic agents is cisplatin. Though used to treat various types of cancer, it has many side effects, and resistance can develop rapidly.^47^ However, its mechanism of action is so universal and efficient that platinum-based analogs are still in use, like carboplatin, oxaliplatin, nedaplatin (second generation), and lobaplatin, heptaplatin (third generation).^48,49^ The resistance of cancer cells to platinum-based anticancer drugs forced scientists to search for other complexes exhibiting cytostatic properties. The medical applications of cobalt complexes, including their anticancer properties, were investigated over the last three decades.^50^ Therefore, we decided to define the ability of the studied cobalt compounds to inhibit cancer cells viability. We also performed cytotoxicity experiments against normal human cells to verify the selectivity of the studied compounds.

The cytotoxicity of selected cobalt complexes was evaluated in three cell lines, two cancer and one normal: human colorectal carcinoma HCT116, human promyelocytic leukemia HL-60, and human fetal lung fibroblast MRC-5 cells. For these preliminary tests, we have selected adhering cells (HCT116) as a model of solid tumors and cells growing in suspension (HL-60) to represent blood cancers. Cells were treated with the following compounds: two molecular Co(ii) C3, C5 and ionic Co(iii) C7, three corresponding ligands (HL1, HL3, HL5), and one reference compound, cisplatin for 72 h, at concentrations ranging from 0.001 to 100 μM. The concentration-dependent inhibition of cell proliferation was observed, and the obtained IC_50_, IC_80_, and IC_90_ values are presented in Table 2. Two out of three studied compounds, C3 and C5, exhibited noticeable and similar cytotoxicity against two cancer cell lines. The sensitivity of HCT116 and HL-60 cells to both compounds did not differ to a significant extent. Calculated IC_50_ of C3/C5 was around 15 μM for HCT116 cells and 19 μM for HL-60. Corresponding ligands, HL1 and HL3, were two times less active than their Co(ii) complexes. Obtained inhibitory concentrations of C3 and C5 are comparable to those presented in the literature for other cobalt complexes.^50–52^ The third selected compound, C7, exhibited lower cytotoxicity against colon cancer and leukemia cells. It was impossible to obtain IC_80_ or IC_90_ doses for this compound, whereas IC_50_ was 71 μM for HCT116 cells and 113 μM for HL-60. In this case, the corresponding ligand HL5 was more active against cancer cells than the complex, especially against leukemia cells. The IC_50_ dose of HL5 for HL-60 cells was calculated as 9 μM, and for HCT116 – 18.5 μM. The viability of HCT116 and HL-60 cells was additionally analyzed upon treatment with a reference compound, cisplatin. Cytotoxicity of cisplatin against cancer cells measured in the same conditions as for cobalt complexes was higher and obtained IC_50_ value did not exceed 0.5 μM.

**Table 2:** Cytotoxicity of selected cobalt complexes against HCT116, HL-60 and MRC-5 cells

Compound	Dose [μM]	Cell line		
		HCT116	HL-60	MRC-5
C3	IC_50_	14.85 ± 7.14	18.97 ± 5.38	20.09 ± 2.06
	IC_80_	34.21 ± 5.73	38.35 ± 2.86	56.78 ± 2.67
	IC_90_	47.16 ± 2.20	49.04 ± 3.55	Not determined
HL1	IC_50_	31.16 ± 0.12	32.63 ± 4.95	39.72 ± 6.62
	IC_80_	70.36 ± 6.20	72.14 ± 13.42	103.76 ± 3.83
	IC_90_	99.90 ± 9.40	101.19 ± 12.70	Not determined
C5	IC_50_	15.25 ± 3.89	18.96 ± 4.11	23.12 ± 1.97
	IC_80_	36.38 ± 2.46	38.62 ± 1.75	57.17 ± 5.55
	IC_90_	48.95 ± 3.42	49.24 ± 1.80	Not determined
HL3	IC_50_	31.15 ± 4.02	37.79 ± 3.62	40.05 ± 4.08
	IC_80_	79.64 ± 10.15	84.70 ± 11.89	119.83 ± 3.73
	IC_90_	109.06 ± 15.57	108.89 ± 14.25	Not determined
C7	IC_50_	70.71 ± 26.01	113.24 ± 24.95	Not determined
	IC_80_	Not determined	Not determined	Not determined
	IC_90_	Not determined	Not determined	Not determined
HL5	IC_50_	18.58 ± 2.96	9.24 ± 0.44	66.15 ± 14.60
	IC_80_	45.91 ± 4.14	35.02 ± 2.23	Not determined
	IC_90_	87.53 ± 18.09	57.34 ± 3.02	Not determined
Cisplatin	IC_50_	0.45 ± 0.04	0.49 ± 0.13	14.67 ± 0.36
	IC_80_	2.65 ± 0.24	2.02 ± 0.79	115.23 ± 11.11
	IC_90_	5.24 ± 0.39	4.89 ± 0.56	Not determined

The cytotoxicity of cobalt complexes and their corresponding ligands was also determined against normal human cells, MRC-5. For all studied compounds and ligands it was difficult to calculate IC_90_ doses, and at least 15% of MRC-5 cells remained alive after 100 μM drug treatment. IC_50_ for C3 was around 20 μM, which means that this compound inhibited the viability of MRC-5 cells to the same level as leukemia cells, HL-60, and lesser than of colon cancer cells, HCT116. MRC-5 cells were slightly less sensitive to the C5 complex than cancer cell lines, with IC_50_ around 23 μM. Both ligands, HL1 and HL3, exhibited the same IC_50_ value, 40 μM against MRC-5 cells and were noticeably less active against normal cells than cancer cells. It is worth emphasizing that for C3, C5, HL1, and HL3 compounds estimated IC_80_ doses against MRC-5 cells were much higher than in the case of cancer cells, which proves that normal cells are slightly less sensitive to these drugs. C7 complex appeared to be almost non-cytotoxic in MRC-5 cells – it was impossible to calculate the IC_50_ dose. For its corresponding ligand, HL5, only IC_50_ value was estimated. It was equal to 70 μM, which was distinctly higher than for both cancer cells (18.5 and 9.2 μM for HCT116 and HL-60 cells, respectively). Importantly, C7 and HL5 compounds at concentrations ranging from 1 to 10 μM, induced the increase of MRC-5 cells viability, which reached even 140% *versus* control. Low cytotoxicity against MRC-5 cells of HL5 and proliferation upregulation caused by this ligand may suggest that the HL5 ligand can exhibit selectivity against normal and cancer cells. We also determined the cytotoxicity of cisplatin against normal MRC-5 cells, which was significantly lower than against cancer cells with the IC_50_ equal to approximately 14.5 μM.

The results considering cancer cells were different from what we have expected *i.e.* higher toxicity of the Co(iii) complex. The situation is however complicated by the distinct character of the Co(iii) complex C7, which is ionic contrary to molecular character of C3 and C5. We suggest that the ionic nature of C7 may impede the transport of the complex into the cancer cells decreasing its anticancer activity. The hypothesis is partly confirmed by the relatively high cytotoxicity of the ligand, which vanishes within the complex; the ligand probably does not enter the cells when bonded to Co(iii) ions. We would like to emphasize that most of the octahedral Co(iii) complexes that were tested for their cytotoxic effects were ionic compounds and they were not very active.^52^ It is important to point out that during the time of incubation of the Co(ii) complexes in the aqueous medium they undergo partial decomposition as verified by TLC experiment described and illustrated in ESI in Fig. 25S† The initial concentrations of the complexes decrease during the experiment, however, there is still a certain amount of the complex in solution after 72 h. Thus the measured effect on cells is due to the contribution of all species that arise during the incubation of the complexes with cells for 72 h.

## Experimental section

### X-ray diffraction and Hirshfeld surfaces

The crystal structure analyses were performed on an STOE IPDS II diffractometer using Mo Kα radiation of a microfocus X-ray source. Crystals were cooled using a Cryostream 800 open flow nitrogen cryostat (Oxford Cryosystems). Data collection and image processing was performed with X-Area 1.75 (STOE & Cie GmbH, 2015).^53^ Intensity data were scaled with LANA (part of X-Area) in order to minimize differences in intensities of symmetry-equivalent reflections (multi-scan method). Structures were solved by direct methods and all non-hydrogen atoms were refined with anisotropic displacement parameters by full-matrix least squares procedure based on F2 using the SHELX-2014 program package.^54^ The Olex^55^ and Wingx^56^ program suites were used to prepare the final version of CIF files. Mercury^57^ was used to prepare the figures. Hydrogen atoms were usually refined using the isotropic model with *U*_iso_(H) values fixed to be 1.5 times *U*eq of C atoms for –CH_3_ or 1.2 times *U*_eq_ for –CH_2_, –NH, and –CH groups. CCDC 2194288–2212180 contain the supplementary crystallographic data for this paper. These data can be obtained free of charge from The Cambridge Crystallographic Data Centre. Crystal data, data collection and structure refinement details are summarized in Table 1S.†

The Hirshfeld surfaces^58^ and 2D fingerprint plots were generated with Crystal Explorer ver. 17.5.^59^

### NMR spectroscopy

NMR spectra (^1^H, ^13^C{^1^H}) of C1A, C1B and HL1–HL5 were recorded on a Bruker AV400 MHz spectrometer (external standard TMS for ^1^H and ^13^C) at ambient temperature in DMSO-*d*_6_. Data were processed using Bruker's Topspin 3.5 software.

### Lipophilicity

The free access web tool SwissADME server (Swiss Institute of Bioinformatics, Lausanne, Switzerland) was used to predict lipophilicity properties of compounds C3, C5 and C7.

### Cytotoxicity studies

To assess the impact of studied complexes on living cells, three cell lines of different type were used: human colorectal carcinoma HCT116 (solid cancer), human promyelocytic leukemia HL-60 (blood cancer) and human fetal lung fibroblast MRC-5 (normal cells). All cell lines were purchased from the American Type Culture Collection (Manassas, VA, ATCC). HCT116 cells, were maintained in McCoy's 5A medium (Merck/Sigma-Aldrich, USA), HL-60 cells in RPMI 1640 medium (Merck/Sigma-Aldrich, USA), while MRC-5 cells in EMEM medium (Merck/Sigma-Aldrich, USA). All media were supplemented with 10% fetal bovine serum (FBS; Biowest, Riverside, MO, USA), while media for cancer cells also with 100 μg mL^−1^ streptomycin, and 100 unit per mL of penicillin. All cells were incubated in 5% CO_2_ atmosphere at 37 °C. Experiments were performed with cells in the exponential phase of growth.

Cytotoxicity was estimated using the MTT method. This is a colorimetric assay, in which cell vability is measured based on metabolic reduction of yellow tetrazole to purple insoluble formazan, what occurs only in living cells. HCT116, HL-60 and MRC-5 cells were seeded in 96-well plates in the number of 2000 per well, 8000 per well, 6000 per well, respectively, and the following day compounds were added at concentrations varing from 0.001 to 100 μM. Stock solutions were prepared as 10 mM in DMSO and dilutions also in DMSO. The final concentration of DMSO in culter medium was 0.5%. Each point was repeated at least 3 times and data were expressed relative to vehicle-treated controls (containing at least 6 points). After 72 h, 3-(4, 5-dimethylthiazol-2-yl)-2,5-diphenyltetrazolium bromi-de (MTT; 80 μg per well) was added for 3 h, plates were centrifuged and supernatants removed. Formazan crystals formed in cells were dissolved in DMSO and absorbance was read at 540 nm. The concentration of the compound required to inhibit cells growth by 50 (IC_50_), 80 (IC_80_) and 90% (IC_90_) compared with untreated control cells was determined from the curves plotting survival as a function of dose. We expressed cytotoxicities with reference to the initial analytical concentration. The growth inhibition assay was conducted at least three times for each compound.

### Syntheses, general remarks

Substrates: 2-hydroxy-3-methoxybenzaldehyde (*o*-vanillin, *o*V), Co(CH_3_COO)_2_·4H_2_O, CoCl_2_·6H_2_O, Et_3_N and aminopyridines (APs) were purchased from commercial sources. Ligands HL1–HL5 were synthesized as described.^18,34,60^ Moreover CoCl_2_·6H_2_O was heated in vacuum to 140 °C for 2 days to obtain an anhydrous salt. Acetonitrile was dried over P_2_O_5_, methanol over magnesium swarf and subsequently both solvents were distilled under argon.

### Reaction between CoCl_2_ and HL1: formation of C1A

The solution of CoCl_2_ (0.032 g, 0.25 mmol) in dry acetonitrile (4 mL) was mixed with the solution of HL1 (0.114 g, 0.5 mmol) in dry acetonitrile (4 mL). The color of the reaction mixture changed from orange to emerald green. After 24 h of stirring at RT, green-brown precipitate formed. Solution was filtered. Filtrate (green solution) was concentrate to half its volume and cooled to +4 °C. After one day, colorless crystals of C1A (*N*-2′′-pyridyl-2,6-dioxo-9-aza-[*c*,*g*]di-2′-methoxybenzo[1.3.3]nonan) were obtained suitable for X-ray diffraction measurement; yield 0.0099 g, 46.9%.

Observing that the solution was green, we assumed that, in addition to the cyclic compound in the reaction mixture there must be also a soluble cobalt complex (C2). In order to identify all products that could form during the reaction, the reaction was repeated according to the procedure described above, and the products were separated by means of preparative TLC in DCM. The fractions were extracted with ethyl acetate, filtered and the obtained filtrates were evaporated to dryness. In this way the following compounds were identify: C1A, C2 (2-aminopyridinium (2-aminopyridine)trichloridocobaltate(II), [Co(2AP)Cl_3_]^−^[2APH]^+^), *o*-vanillin, 2-aminopyridine and unreacted HL1. C2 (emerald-green crystals), which structure was published before,^42^ was identified by X-ray diffraction analysis. The remaining components of the reaction mixture were identified by their FT-IR spectra (comparison with the FT-IR spectra of the pure compounds).

The same reaction was also repeated with the different time, temperature and finally with the change of the solvent:

• Time of the reaction elongated to 48 h. Yield of C1A: 0.0282 g, 66.9%.

• The change in the temperature from RT to 40 °C, time 24 h. Yield of C1A: 0.0031 g, 7.3%; C2: 0.0216 g, 51.2%. Higher temperature resulted in an increase in the yield of formation of ionic pair.

• The change of solvent to anhydrous methanol. The reaction did not occur. The unchanged ligand HL1 was present in the reaction mixture.

Elemental analysis (C1A): anal. calcd. for C_21_H_18_N_2_O_4_: C 69.6; H 5.01; and N 7.73, anal. found C 69.58; H 5.02; and N 7.72.

FT-IR (C1A): 3089(vw), 3065(vw), 3053(w), 3052(w), 3051(w), 3008(w), 2955(w), 2954(w), 2934(w), 2904(w), 2835(w, sh), 1898(vw), 1846(vw), 1589(s), 1569(s), 1486(s), 1474(vs), 1436(vs, sh), 1399(m, sh), 1348(m), 1329(m), 1310(s), 1287(s), 1261(vs), 1229(vs), 1228(vs), 1216(vs), 1199(vs), 1176(s), 1154(s), 1108(s), 1096(s), 1086(s), 1074(s), 1055(m), 1015(s), 988(s), 980(s), 957(s), 924(s), 883(s), 882(s), 816(w), 814(w), 792(m, sh), 770(m), 760(s), 750(s), 729(s), 726(s), 710(m), 679(m), 626(m), 614(m), 588(m), 570(w), 569(w), 544(w), 543(w), 520(w), 514(w), 492(w), 468(w), 456(w), 405(w,sh) cm^−1^.

^1^H NMR of C1A (DMSO-*d*_6_): 8.23 (dd, *J* = 4.9, 1.2 m Hz, 1H, o,m,p-CH); 7.74 (ddd, *J* = 8.9, 7.3, 1.9 Hz, 1H, o,m,p-CH); 7.41–7.34 (m, 3H, o,m,p-CH); 7.13–7.06 (m, 2H, N–CH–O); 6.95–6.87 (m, 5H, o,m,p-CH); 3.70 (s, 6H, O–CH_3_) ppm.

^13^C{^1^H} NMR of C1A (DMSO-*d*_6_): 154.44; 148.37; 148.18; 141.58; 139.14; 121.15 (d, *J* = 5.3 Hz); 120.96, 117.21; 113.29; 109.98; 76.41; 55.93; 41.18–39.10 (m) ppm.

### Reaction between CoCl_2_ and HL2

The solution of CoCl_2_ (0.019 g, 0.15 mmol) in dry acetonitrile (2 mL) was mixed with the solution of HL2 (0.068 g, 0.3 mmol) in dry acetonitrile (2 mL). The color of the reaction mixture changed from orange to vivid green. After 24 h of stirring at RT, green precipitate formed. Solution was filtered. By TLC in DCM we identified that the Schiff base, complex, *o*-vanillin and 3-aminopyridine were present in the reaction mixture. The reaction was repeated with the changed of the solvent to DCM and the volume increased from 4 mL to 40 mL. Crystal formation was observed at +4 °C. By X-ray diffraction analysis we confirmed the presence of an ion pair [Co(3AP)Cl_3_]^−^[3APH]^+^. The crystals were of very low quality (*R*_int_ = 40%) and we do not include the crystal structure in this paper. The tentative parameters of triclinic unit cell at 120 K were: *a* = 7.681(17), *b* = 14.30(3), *c* = 14.66(3), *α* = 104.65(16)°, *β* = 105.12(15)°, *γ* = 105.30(16)°. We did not observe the formation of cyclic compound.

### Reaction between CoCl_2_ and HL3: formation of C1B

The solution of CoCl_2_ (0.128 g, 1.0 mmol) in dry acetonitrile (12 mL) was mixed with the solution of HL3 (0.484 g, 2.0 mmol) in dry acetonitrile (12 mL). The color of the reaction mixture changed from orange to dark green. After 48 h of stirring at RT, green precipitate formed. Solution was filtered. Filtrate was concentrate to half its volume and cooled to +4 °C. After one day, pale-green crystals of C1B were obtained; yield 0.045 g, 12%. The yield was not optimized. The crystal structure of C1B was determined by X-ray analysis. (ESI Fig. 1S†). Identification of all products of the reaction was performed by preparative TLC in DCM. The fractions were extracted with ethyl acetate, filtered and the obtained filtrates were evaporated to dryness. The following compounds were identified on the basis of their FT-IR ATR spectra: C1B, [Co(2APMe4)Cl_3_]^−^[2APHMe]^+^, *o*-vanillin, 2-amino-4-methylpyridine and unreacted HL3.

Elemental analysis (C1B): anal. calcd. for C_22_H_20_N_2_O_4_: C 70.2; H 5.36; and N 7.44, anal. found C 69.89; H 5.36; and N 7.57.

FT-IR (C1B): 3098.90(w), 3056.51(w), 3010.46(w), 3006.20(w), 2970.16(w), 2947.04(w), 2925.92(w), 2860.45(w), 2840.05(w), 2788.96(w), 2705.59(w), 2582.62(w), 2493.00(w), 2115.33(vw), 2088.97(vw), 2062.12(vw), 1996.28(vw), 1908.26(vw), 1824.47(vw), 1750.04(vw), 1662.24(vw), 1594.93(vs), 1576.20(m), 1547.88(m), 1459.67(vs), 1439.52(s), 1417.11(s), 1401.81(m), 1373.74(m), 1373.74(m), 1344.50(w), 1331.32(w), 1277.91(w), 1246.56(vs), 1218.02(s), 1173.36(m), 1147.34(s), 1112.42(w), 1094.42(m), 1074.55(m), 994.85(w), 968.26(m), 938.24(w), 880.13(w), 854.57(w), 840.24(w), 812.75(w), 801.22(w), 782.50(w), 744.59(w), 732.35(m), 664.87(w), 602.37(vw), 586.53(w), 572.82(vw), 544.11(w), 532.98(vw), 518.31(w), 503.18(vw), 446.84(w) cm^−1^.

^1^H NMR of C1B (DMSO-*d*_6_): 8.08 (d, *J* = 5,1 Hz, 1H, o,m,p-CH); 7.36 (s, 2H, o,m,p-CH); 7.24 (s, 1H, o,m,p-CH); 7.12–7.08 (m, 2H, N–CH–O); 6.92 (s, 2H, o,m,p-CH); 6.91 (d, *J* = 1.5 Hz, 2H, o,m,p-CH); 6.76 (d, *J* = 5.1 Hz, 1H, o,m,p-CH); 3.70 (s, 6H, O–CH_3_); 2.29 (s, 3H, C–CH_3_) ppm.

^13^C{^1^H} NMR of C1B (DMSO-*d*_6_): 154.44; 148.37; 148.18; 141.58; 139.14; 121.15 (d, *J* = 5.3 Hz); 120.96, 117.21; 113.29; 109.98; 76.41; 55.93; 41.18–39.10 (m) ppm.

### Synthesis of Co(L1)_2_, C3

The methanolic solution (10 mL) of HL1 (0.162 g, 0.7 mmol) was mixed with the methanolic solution (10 mL) of Co(CH_3_COO)_2_·4H_2_O (0.088 g, 0.35 mmol). The reaction mixture was stirred for 30 minutes. Then to the mixture, 0.07 mL Et_3_N (0.5 mmol, 0.726 g dm^−3^) was added. The reaction mixture changed color from orange to brownish-red. Crystals suitable for X-ray diffraction analysis were obtained at +4 °C. Yield: 0.0768 g, 42%.

Elemental analysis: anal. calcd. for C_26_H_22_N_4_CoO_4_: C 60.82; H 4.32; and N 10.91, anal. found C 60.48; H 4.488; and N 11.06.

FT-IR: 3079.49(vw), 3044.79(w), 2996.63(w), 2951.60(w), 2924.96(w), 2900.52(w), 2826.63(w), 2615.37(w), 2081.54(vw), 1979.71(vw), 1882.88(vw), 1766.62(vw), 1602.32(s), 1583.59(s), 1561.46(s), 1537.91(s), 1462.89(m), 1446.00(m), 1418.66(vs), 1399.80(s), 1381.08(s), 1330.45(s), 1302.42(m), 1278.05(vw), 1235.94(s, sh), 1182.48(vs), 1165.30(s), 1152.24(s), 1104.60(m), 1077.44(m), 1050.61(m), 981.30(m), 881.73(m), 854.06(m), 789.05(m), 778.57(m), 736.66(m), 658.39(w), 635.1(w), 612.05(w), 579.25(w), 567.14(w), 551.47(m), 504.91(w), 425.53(w), 411.23(w) cm^−1^.

### Synthesis of [Co(L2)_2_], C4

The methanolic solution (20 mL) of HL2 (0.114 g, 0.5 mmol) was mixed with the methanolic solution (20 mL) of Co(CH_3_COO)_2_·4H_2_O (0.062 g, 0.25 mmol). The reaction mixture was stirred for 30 minutes. Then 0.07 mL of Et_3_N (0.5 mmol, 0.726 g dm^−3^) was added. After five minutes, a yellow-brown precipitate began to form. The precipitate was collected after 10 min of further stirring and then washed with a small amount of cold Et_2_O. Yield: 0.1003 g, 78%.

Elemental analysis: anal. calcd. for C_26_H_24_N_4_CoO_4_: C 60.59; H 4.69; and N 10.87, anal. found C 60.04; H 4.392; and N 11.04.

FT-IR: 3048.87(w), 2997.38(w), 2976.05(w), 2948.37(w), 2930.03(w), 2889.98(w), 2829.49(w), 2591.52(vw), 1980.26(vw), 1738.18(vw), 1604.68(vs, sh), 1577.52(s), 1539.52(s, sh), 1465.27(vs, sh), 1444.43(vs, sh), 1420.43(s), 1388.27(m), 1357.18(w), 1337.43(m), 1321.36(m), 1232.12(vs, sh), 1197.66(vs), 1184.85(vs), 1103.06(m), 1078.22(m), 1046.41(m), 1029.30(m), 979.12(m), 961.39(w), 869.33(w), 852.45(m), 810.00(m, sh), 785.90(w), 736.31(m, sh), 707.22(m, sh), 657.56(w), 472.66(w), 411.71(w) cm^−1^.

### Synthesis of Co(L3)_2_, C5

The solution of HL3 (0.242 g, 1 mmol) in a mixture of solvents: DMSO (7 mL) and acetonitrile (5 mL) was mixed with the solution of Co(CH_3_COO)_2_·4H_2_O (0.125 g, 0.5 mmol) dissolved in 12 mL DMSO/acetonitrile (7 : 5). The reaction mixture was stirred for 15 minutes. Then to this solution 0.07 mL Et_3_N (0.5 mmol, 0.726 g dm^−3^) was added. A red precipitate has formed. The precipitate of C5 was collected after 10 min of further stirring and then washed with a small amount of cold Et_2_O. Yield: 0.1201 g, 45%.

Initially reaction between HL3 and cobalt acetate was carried out in methanol, which also leads to the formation of C5. Due to low yield of the synthesis in methanol – up to 10% (0.0024 g), the solvent has been changed from methanol to DMSO/acetonitrile.

Crystals suitable for X-ray diffraction analysis were obtained at +4 °C from methanol.

Elemental analysis: anal. calcd. for C_28_H_26_N_4_CoO_4_: C 62.11; H 4.84; and N 10.35, anal. found C 61.48; H 4.793; and N 10.25.

FT-IR: 3084.75(vw), 3034.75(w), 2995.83(w), 2958.74(w), 2930.84(w), 2916.97(w), 2832.25(w), 2728.78(vw), 2648.15(vw), 2609.18(vw), 2088.98(vw), 2030.77(vw), 1976.18(vw), 1868.20(vw), 1799.58(vw), 1605.25(s, sh), 1587.26(s), 1577.39(s), 1566.94(s), 1537.37(s, sh), 1480.12(m), 1465.87(m), 1426.06(vs), 1416.74(vs), 1371.28(s), 1337.92(m), 1324.09(m), 1298.46(w), 1275.65(w), 1259.14(vw), 1234.80(s), 1220.25(s), 1187.67(s), 1175.94(vs), 1139.08(s), 1108.87(m), 1078.68(m), 1048.41(m), 1024.53(m), 981.31(m), 929.65(w), 898.80(vw), 885.98(w), 842.46(w), 830.73(m), 824.48(m), 798.85(vw), 729.72(m), 654.08(w), 629.57(vw), 606.72(w, sh), 579.61(vw), 568.31(w), 541.59(w), 517.93(vw), 508.04(w), 470.63(vw), 421.30(w) cm^−1^.

### Synthesis of Co(L4)_2_(OV)_2_ ethanol, C6

The ethanolic solution (8 mL) of HL4 (0.242 g, 1.0 mmol) was mixed with the ethanolic solution (8 mL) of Co(CH_3_COO)_2_·4H_2_O (0.125 g, 0.5 mmol). The reaction mixture was stirred for 5 minutes. Then 0.07 mL of Et_3_N (0.5 mmol, 0.726 g dm^−3^) was added. Crystals suitable for X-ray diffraction analysis were obtained at RT, by slow evaporation of the solvent. Yield: 0.1305 g, 23%.

Elemental analysis: anal. calcd. for C_50_H_54_N_4_Co_3_O_15_: C 53.25; H 4.83; and N 4.97, anal. found C 53.48; H 4.88; and N 4.97.

FT-IR: 3419.97(vw), 3114.56(vw), 3063.21(vw), 3018.54(vw), 2964.06(w), 2936.7(vw), 2893.35(vw), 2856.84(vw), 2838.59(vw), 2767.69(vw), 2573.02(vw), 2494.11(vw), 2324.35(vw), 1682.95(vw), 1655.31(m), 1601.48(vs), 1568.00(s), 1550.02(s), 1479.88(vs), 1468.75(s), 1438.72(vs), 1391.34(s), 1384.98(s), 1308.10(m), 1297.80(w), 1274.41(m), 1240.70(m), 1211.20(vs), 1176.43(s), 1159.39(m), 1099.56(w), 1075.91(m), 1058.74(w), 1035.80(m), 1019.62(w), 979.85(w), 964.92(w), 944.54(w), 907.20(w), 856.79(w), 844.96(w), 827.76(w), 802.24(w), 782.44(w), 771.23(w), 750.45(m), 741.15(m), 730.63(w), 643.38(w), 616.42(w), 594.56(w), 560.65(w), 540.18(w), 502.70(w), 473.86(w), 456.27(w), 446.41(w) cm^−1^.

### Synthesis of Et_3_NH^+^[Co(L5)_2_]^−^ methanol, water solvate, C7

The methanolic solution (10 mL) of HL5 (0.245 g, 1.0 mmol) was mixed with the methanolic solution (6 mL) of Co(CH_3_COO)_2_·4H_2_O (0.125 g, 0.5 mmol). The reaction mixture was stirred for 10 minutes. Then 0.14 mL of Et_3_N (1.0 mmol, 0.726 g dm^−3^) was added. After five minutes, a brown precipitate started to form. After 30 minutes of stirring, the disappearance of the precipitate could be observed. Crystals suitable for X-ray diffraction analysis were obtained at +4 °C. Yield: 0.1324 g, 38%.

Elemental analysis: anal. calcd. for C_35_H_44_N_3_CoO_8_: C 60.60; H 6.39; and N 6.06, anal. found C 58.87; H 6.138; and N 5.83.

FT-IR: 3607.88(m), 3462.73(s), 3307.20(m), 3152.81(s), 3050.38(s), 3029.41(s), 2991.15(s), 2927.42(s), 2900.05(m), 2818.11(m), 2666.68(m), 2505.55(m), 1921.62(vw), 1888.11(vw), 1850.42(vw), 1723.13(vw), 1644.18(vw), 1612.03(s), 1600.72(s), 1582.64(s), 1537.83(s), 1478.76(vs), 1458.49(s), 1433.06(vs), 1391.01(vs), 1330.36(m), 1300.67(vs), 1274.91(s), 1228.37(vs), 1174.29(vs), 1144.34(s), 1107.79(s), 1080.27(s), 1046.18(m), 1030.05(m), 982.68(m), 972.30(m), 931.39(w), 887.95(w), 858.27(w), 836.26(m), 789.56(vw), 777.46(vw), 749.80(m), 730.98(s), 668.89(w), 642.74(w), 626.16(w), 578.91(w), 560.58(vw), 531.85(m), 456.37(vw), 442.53(vw), 428.47(vw) cm^−1^.

## Conclusions

We have observed the cyclic condensation of HL1 and HL3, which was catalyzed by the presence of cobalt(ii) chloride in dry acetonitrile. The 2-aminopyridine/2-amino-4-methylpyridine eliminated during the self-cyclization forms a complex cation with cobalt(ii) ions. The cyclization was observed in non-aqueous conditions. Cobalt(ii) acetate in methanol reacts with hemisalen ligands HL1–HL4 forming Co(ii)-imine complexes of diverse nuclearity. The reaction of cobalt(ii) acetate with bisphenol HL5 leads to the spontaneous oxidation of Co(ii) and the formation of the cobalt(iii) complex. We suggest that this ligand and other bisphenolates strongly stabilize the (+3) oxidation number of cobalt.

For the selected molecular Co(ii) complexes C3, C5, and ionic Co(iii) complex C7, cytotoxicity studies towards cancer and normal cells were performed. Among the selected complexes, we expected higher toxicity of the Co(iii) complex due to its higher inertness.^61^ However, the cytotoxicity of C7 was the lowest among the studied compounds, despite the fact that imine HL5, used to synthesize compound C7, exhibited the highest cytotoxicity. The results can be explained either by the ionic character of C7, which hinders its transport into the cancer cells, or by the instability of the studied complexes in the aqueous media. All studied compounds: ligands and complexes are slightly less active against normal cells than cancer cells. The HL5 ligand showed cytotoxicity towards cancer cells and induced growth of the normal cells, which makes it the most selective out of the studied compounds: hemisalens and their complexes with cobalt.

## Conflicts of interest

There are no conflicts to declare.

## Supplementary Material

RA-013-D2RA07089H-s001

RA-013-D2RA07089H-s002
