# Supplementary material for: Reactions of cobalt(ii) chloride and cobalt(ii) acetate with hemisalen-type ligands: ligand transformation, oxidation of cobalt and complex formation. Preliminary study on the cytotoxicity of Co(ii) and Co(iii) hemisalen complexes
Source: RSC Adv. 2023 Mar 16;13(13):8830–43. doi: 10.1039/d2ra07089h (PMC10018370; doi:10.1039/d2ra07089h)
Supplement: RA-013-D2RA07089H-s001 [file RA-013-D2RA07089H-s001.pdf]

Electronic Supplementary Material (ESI) for RSC Advances.
This journal is © The Royal Society of Chemistry 2023

Reactions of cobalt(II) chloride and cobalt(II) acetate with hemisalen-type imines: ligand transformation, oxidation of cobalt and complex formation. Preliminary study on the cytotoxicity of Co(II) and Co(III) hemisalen complexes

Magdalena Siedzielnik[a]*, Monika Pawłowska[b], Mateusz Daśko[a], Hubert Kleinschmidt[a] and Anna Dołęga[a]

[a.] *Department of Inorganic Chemistry, Chemical Faculty, Gdansk University of Technology, Narutowicza 11/12, 80-233 Gdansk, Poland*

[b.] *Department of Pharmaceutical Technology and Biochemistry, Chemical Faculty, Gdansk University of Technology, Narutowicza 11/12, 80-233 Gdansk, Poland*

Supporting Information

Table of Contents

1. Crystallographic details and crystal structure of **C1B** and **C2**
2. Experimental and theoretical structure of **C3**
3. The plausible mechanism of the self-cyclization reaction of **HL1**
4. NMR spectra ^{1}H, $^{13}C\{^{1}H\}$ of **C1A**, **C1B** and NMR spectra ^{1}H of **HL1**-**HL5**
5. FT-IR spectra of **C1A**-**C7**
6. TLC of **C5**

1. **Crystallographic details and crystal structures of C1B and C2**

Table 1S. Crystallographic data for compounds **C1A, C1B, C2, C3**, and **C5-C7**

Compound	C1A	C1B	C2	C3	C5	C6	C7
Empirical formula	$C_{21}H_{18}N_2O_4$	$C_{22}H_{20}N_2O_4$	$C_{10}H_{13}Cl_3CoN_4$	$C_{26}H_{22}CoN_4O_4$	$C_{28}H_{26}CoN_4O_4$	$C_{52}H_{58}Co_3N_4O_{16}$	$C_{35}H_{44}CoN_3O_8$
Formula weight	362.37	376.4	354.52	513.4	541.46	1171.81	693.66
Wavelength [Å]	0.71073	0.71073	0.71073	0.71073	0.71073	0.71073	0.71073
T [K]	120	120	120	120	120	120	120
Crystal system	Triclinic	Triclinic	Monoclinic	Monoclinic	Monoclinic	Monoclinic	Monoclinic
Space group	*P*-1	*P*-1	*Cc*	$P2_1/n$	$P2_1/c$	$P2_1/n$	$P2_1/c$
a (Å)	9.2880(5)	8.9262(4)	12.9563(11)	12.5620(10)	13.6519(4)	10.9556(8)	11.359(3)
b (Å)	9.6491(5)	9.3131(4)	8.0625(4)	15.3891(8)	13.7225(5)	20.9941(12)	11.974(7)
c (Å)	10.2198(5)	11.4785(5)	14.0076(17)	12.9050(12)	26.1260(8)	11.8225(9)	24.537(7)
α (°)	66.574(4)	87.538(4)	90.00	90.00	90.00	90.00	90.00
β (°)	82.570(4)	85.441(4)	106.127(8)	114.666(7)	90.899(3)	97.027(6)	99.62(2)
γ (°)	84.516(4)	68.748(3)	90.00	90.00	90.00	90.00	90.00
V (Å^3)	832.37(8)	886.41(7)	1405.7(2)	2267.1(4)	4893.8(3)	2698.8(3)	3291(2)
Z	2	2	4	4	4	2	4
D_c (g·cm^{-3})	1.446	1.41	1.675	1.504	1.47	1.442	1.4
μ(mm^{-1})	0.101	0.10	1.777	0.80	0.745	0.98	0.579
F (000)	380	396	716	1060	2248	1214	1464
Reflection collected	10107	12586	11141	18864	52634	28607	26441
Unique reflections	4447	4767	3605	6107	13225	7312	8840
Parameters	246	256	183	318	675	346	441
R_{int}	0.026	0.018	0.027	0.030	0.052	0.053	0.079
GOOF	1.084	1.04	1.048	1.062	1.027	1.034	0.997
$R_1[I>2\sigma(I)]$	0.037	0.038	0.026	0.031	0.056	0.057	0.057
wR_2 (all data)	0.1046	0.1015	0.069	0.081	0.129	0.161	0.1687
CCDC numbers	**2194288**	**2237609**	**2194289**	**2194290**	**2194291**	**2212180**	**2194292**

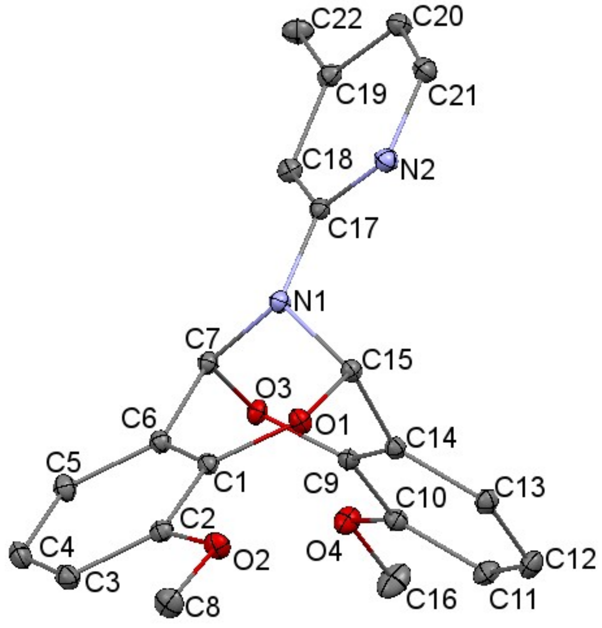

Figure 1S. Molecular structure of **C1B** with the numbering scheme; important bond lengths [Å]: N1—C17 1.4074(12), N1—C7 1.4338(12), N1—C15 1.4556(12); important angles [°]: C17—N1—C7 120.76(8), C17—N1—C15 117.37(8), C7—N1—C15 108.74(7)

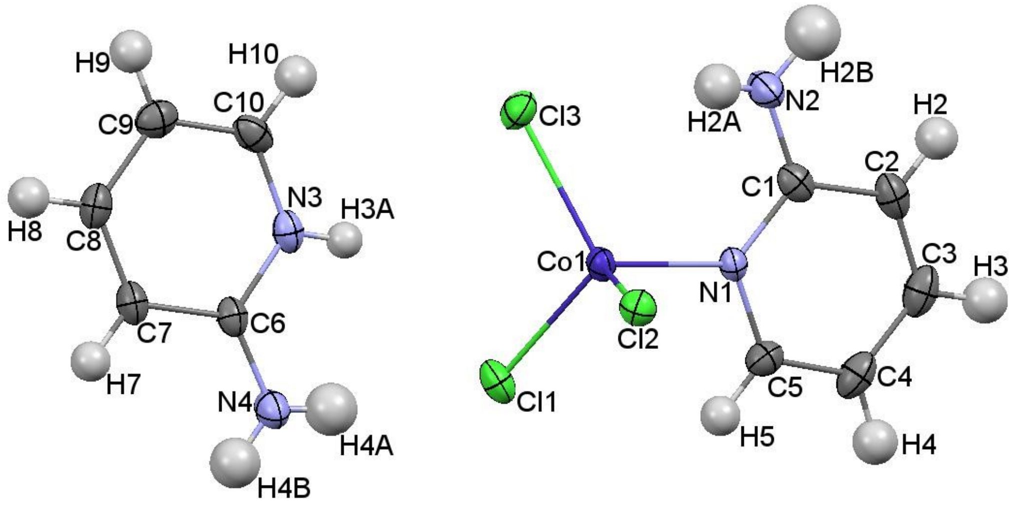

Figure 2S. Molecular structure of **C2** with the numbering scheme; important bond lengths [Å]: Co1—N1 2.031(2), Co1—Cl1 2.2719(8), Co1—Cl2 2.2644(7), Co1—Cl3 2.2626(8); important angles [°]: N1—Co1—Cl3 113.81(7), N1—Co1—Cl2 109.13(7), Cl3—Co1—Cl2 107.97(3), N1—Co1—Cl1 104.86(7), Cl3—Co1—Cl1 108.52(3), Cl2—Co1—Cl1 112.64(3)

2. Experimental and theoretical structure of **C3**

For the details of the DFT calculations please see the ref. 39 M. Siedzielnik, D. A. Pantazis, J. Bruniecki, K. Kaniewska-Laskowska and A. Dołęga, Crystals (Basel), 2021, **11**, 1–15.

Table 2S X-ray experimental and DFT calculated geometrical parameters of complex **C3**

	Experimental values	DFT calculated values
Bond lengths / contacts [Å]		
Co1–N1	2.0108(12)	1.990
Co1–N3	1.9990(12)	1.991
Co1–O1	1.9255(10)	1.905
Co1–O3	1.9389(10)	1.939
Co1---N4	2.711(1)	2.791
Bond angles [°]		
N1–Co1–N3	128.54(5)	120.95
N1–Co1–O1	95.09(4)	96.98
N1–Co1–O3	111.24(5)	114.35
N3–Co1–O1	118.00(5)	118.56
N3–Co1–O3	91.08(5)	93.11
O1–Co1–O3	113.81(5)	114.83

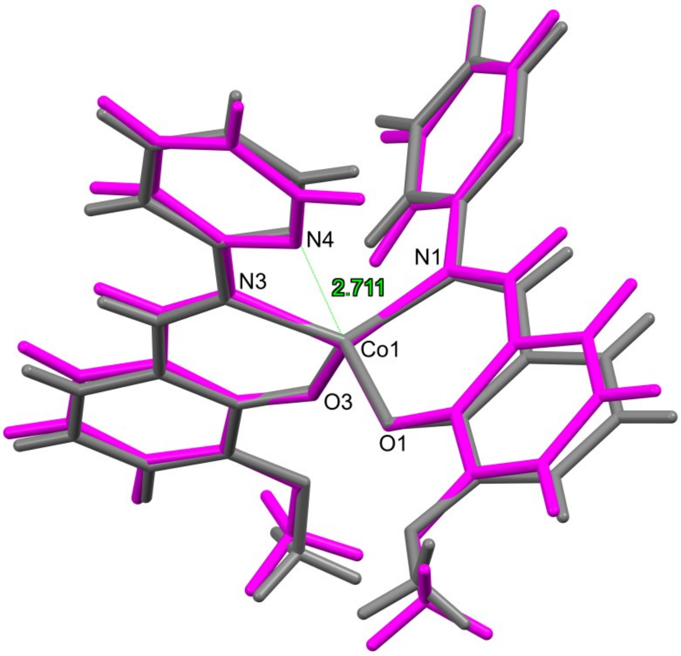

Figure 3S. The overlay of the experimental (grey) and DFT calculated molecular structure of **C3**. The overlay was performed in program Mercury for Co1 and its coordination sphere, *i.e.* atoms: N1, N3, O1, O3. Experimental contact Co1---N4 indicated.

3. The plausible mechanism of the self-cyclization reaction of **HL1**

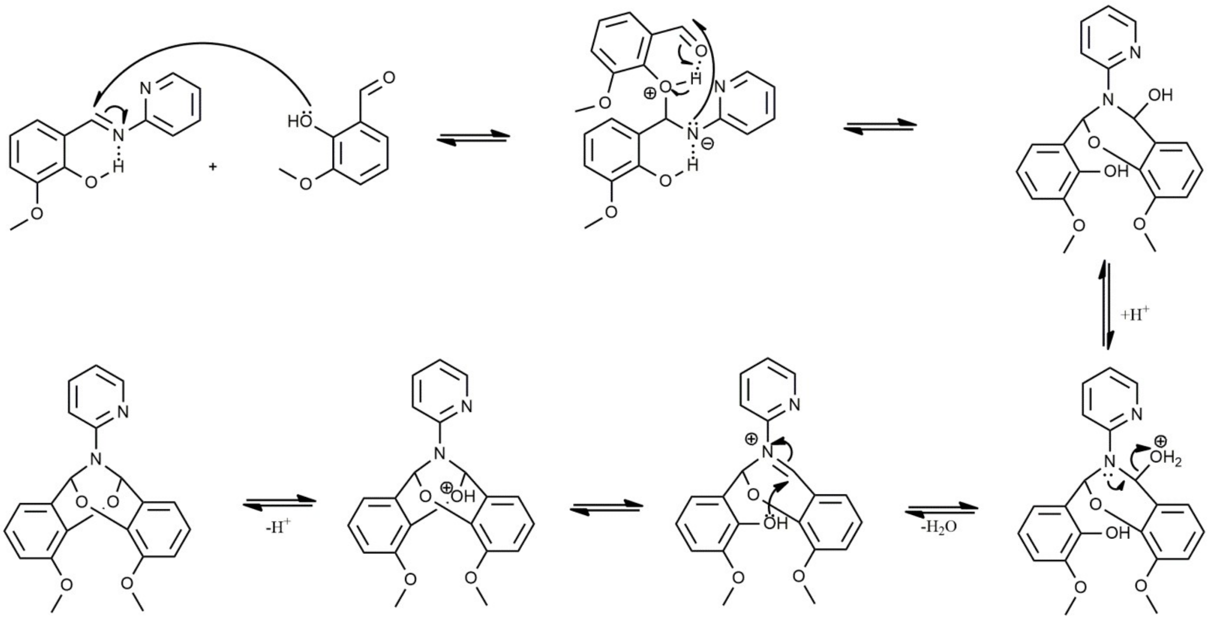

Scheme 1S. The alternative mechanism of cyclisation of imine **HL1** with the participation of the product of hydrolysis – o-vanillin.

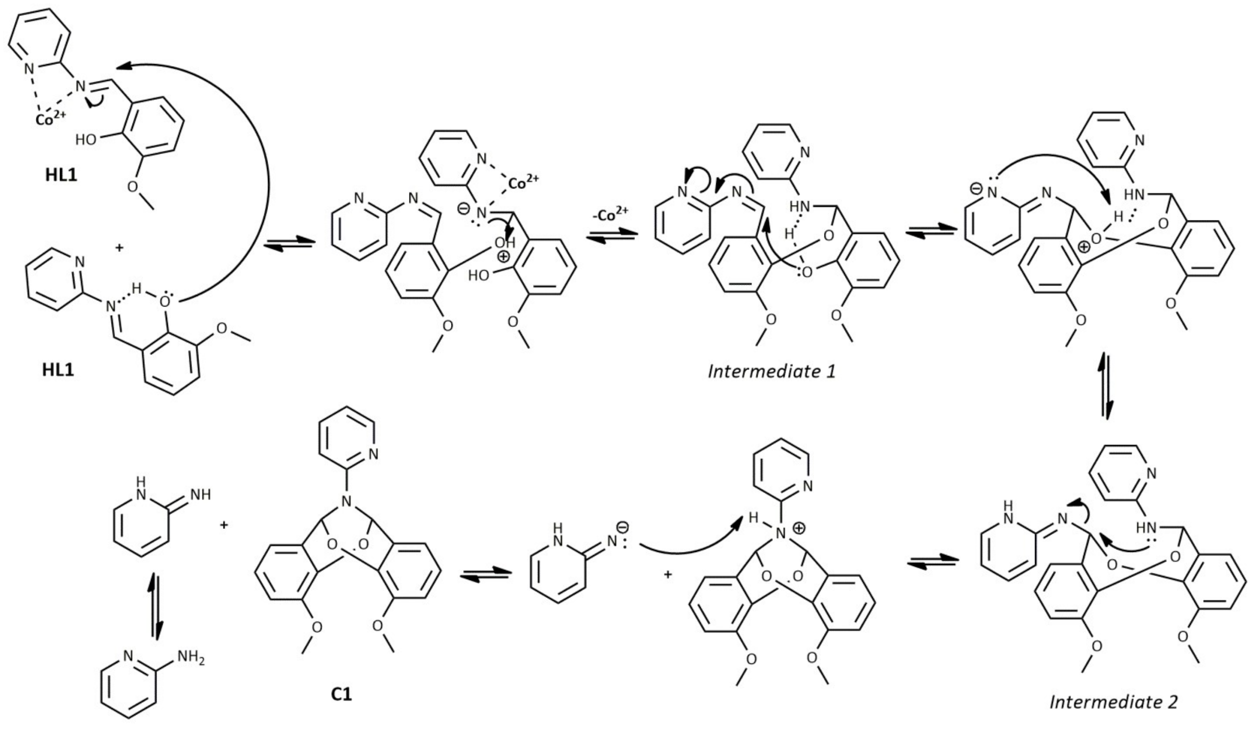

Scheme 2S. The mechanism of cyclisation of imine **HL1** with the participation of Co(II) ions.

4. NMR spectra of **C1A** and **C1B** and **HL1**-**HL5**

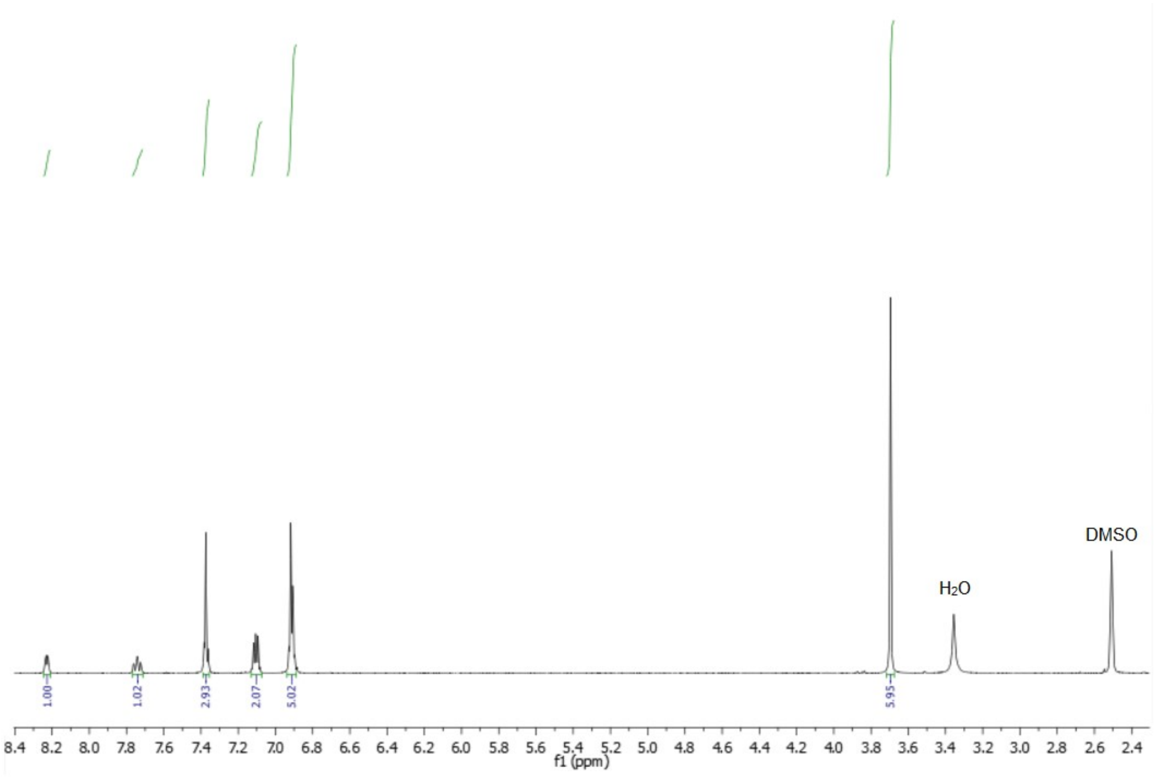

Figure 4S. ^{1}H NMR of **C1A** at room temperature in DMSO-d6

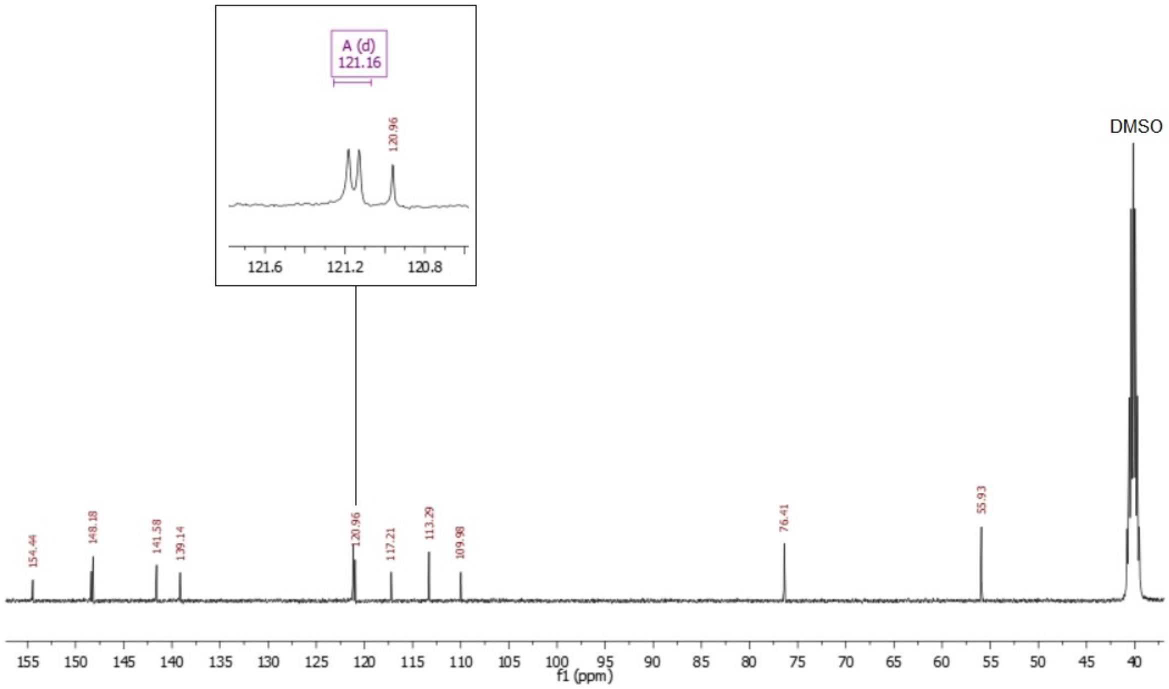

Figure 5S. $^{13}C\{^{1}H\}$ NMR of **C1A** at room temperature in DMSO-d6

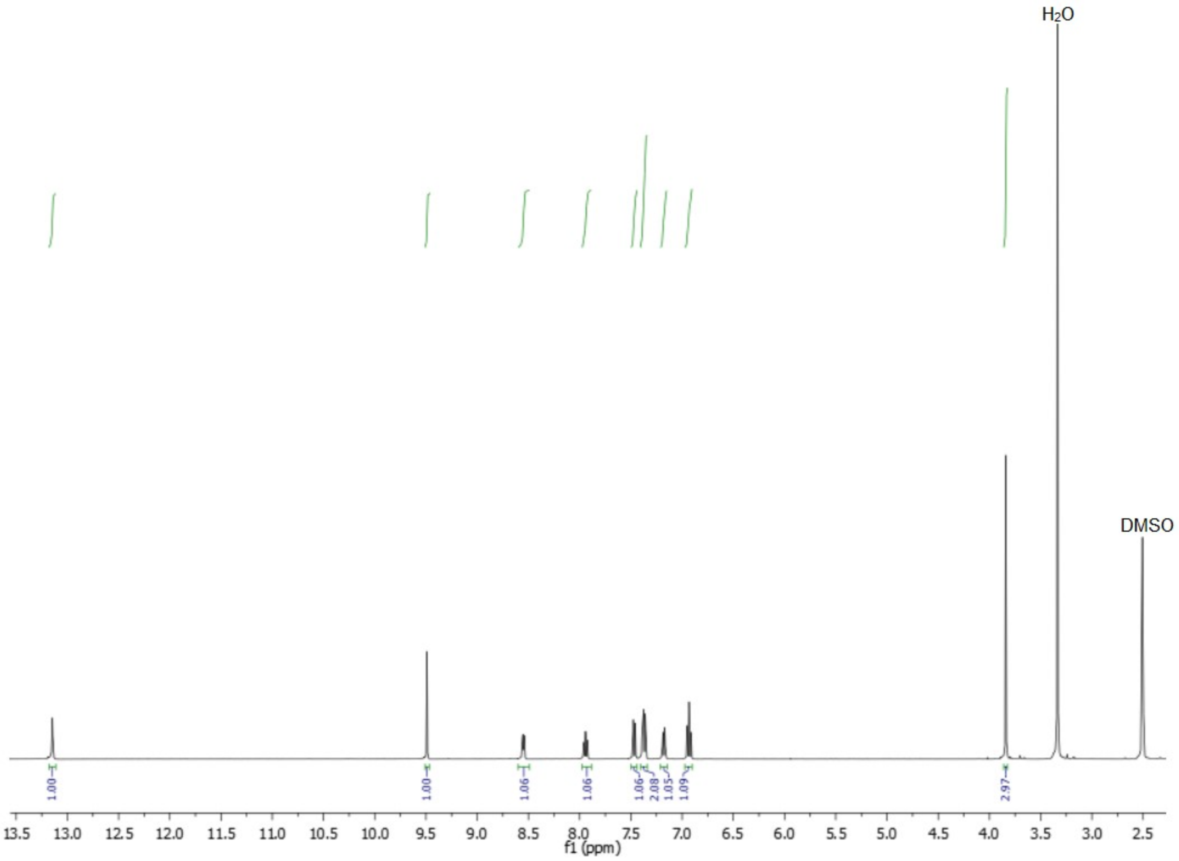

Figure 6S. ^{1}H NMR of **HL1** at room temperature in DMSO-d6

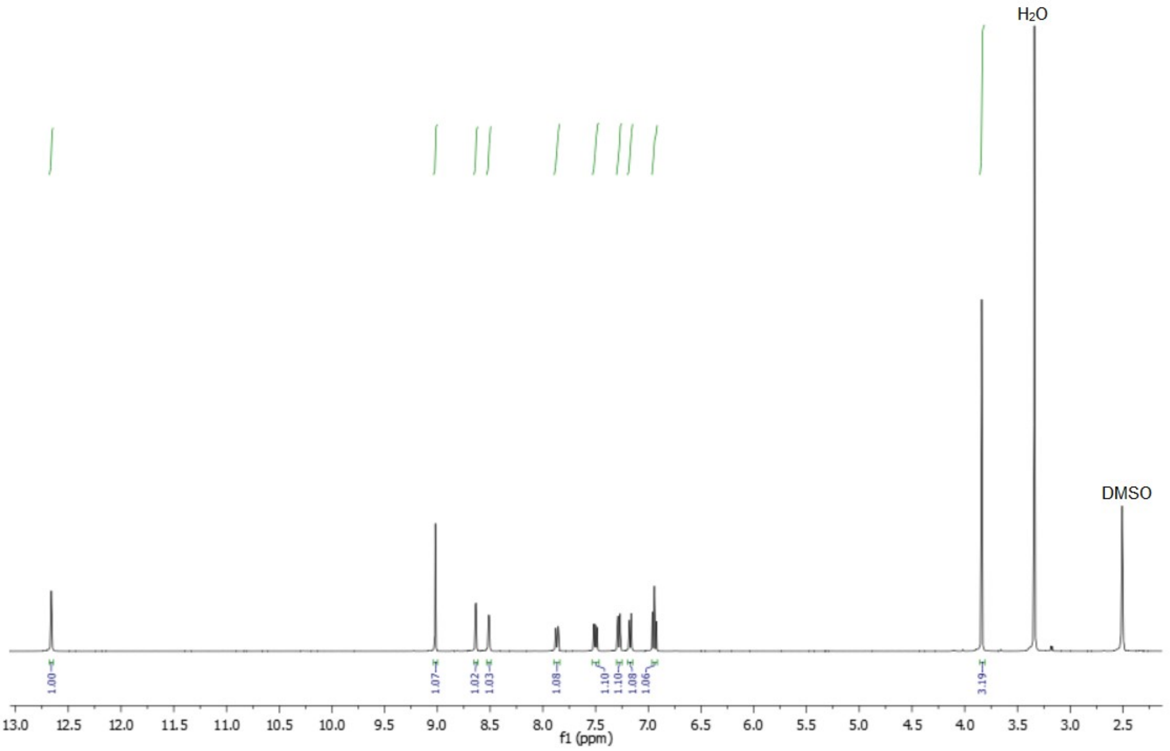

Figure 7S. ^{1}H NMR of **HL2** at room temperature in DMSO-d6

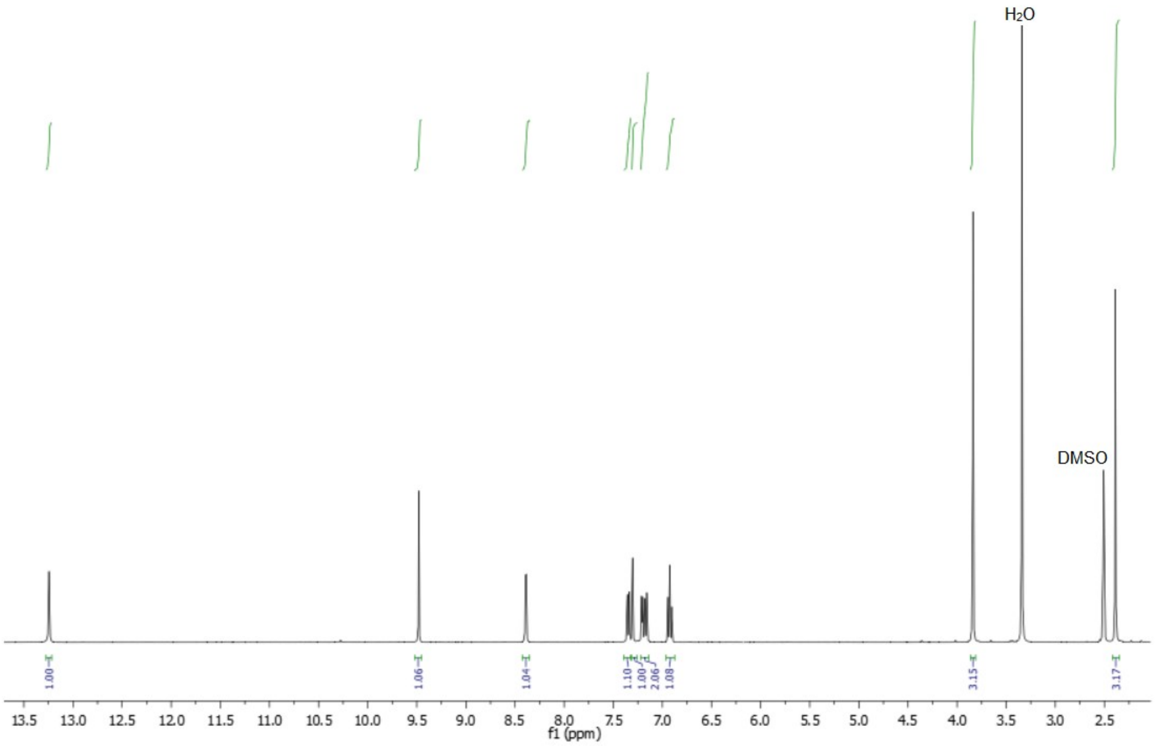

Figure 8S. ^{1}H NMR of **HL3** at room temperature in DMSO-d6

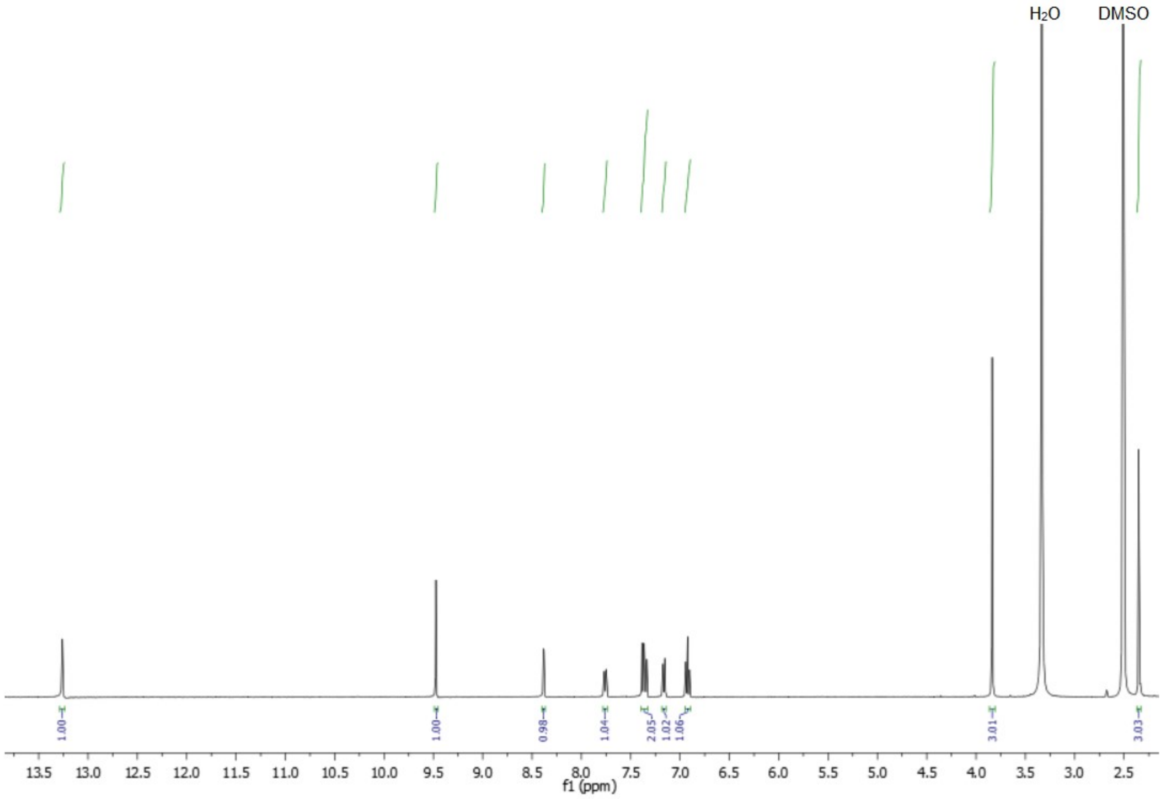

Figure 9S. ^{1}H NMR of **HL4** at room temperature in DMSO-d6

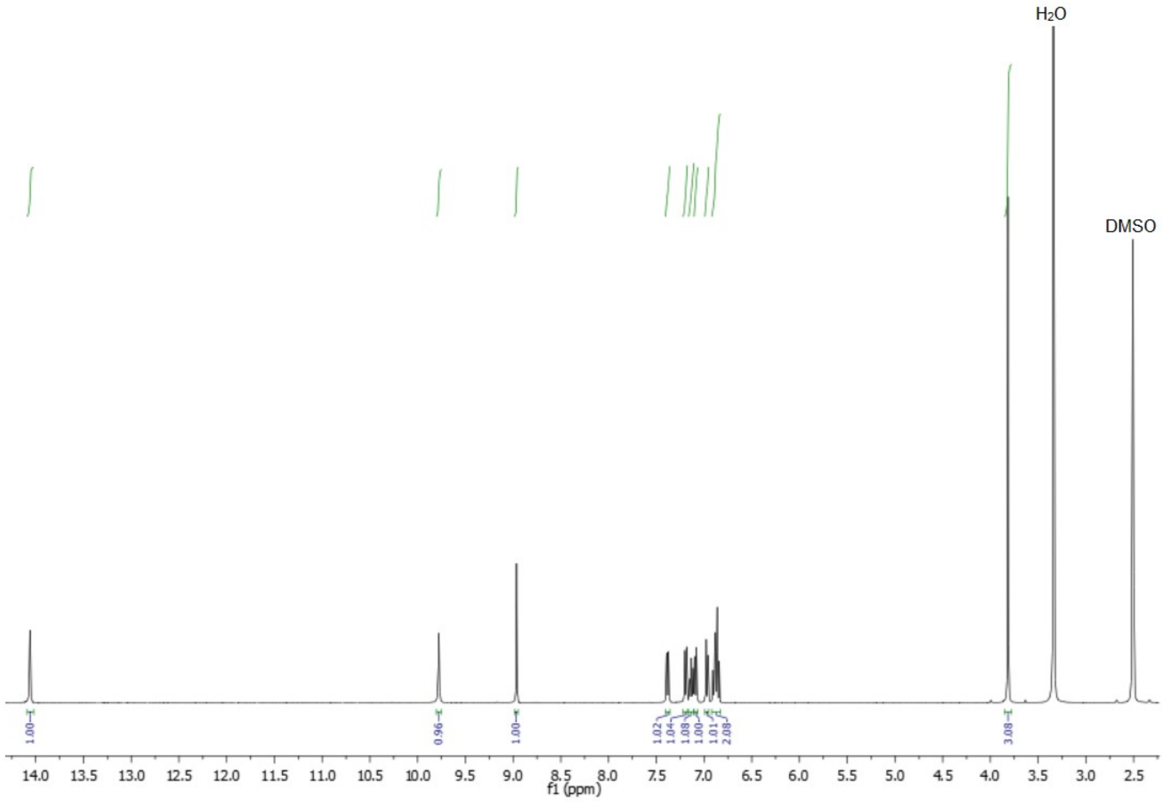

Figure 10S. ^{1}H NMR of **HL5** at room temperature in DMSO-d6

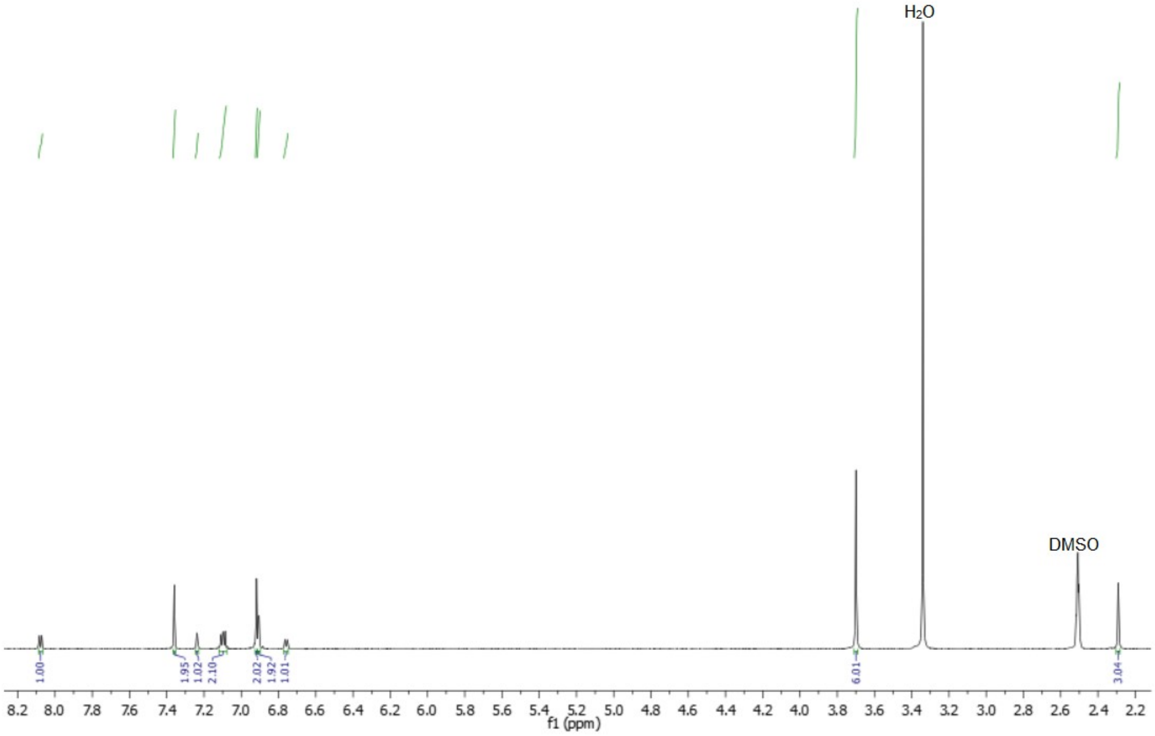

Figure 11S. ^{1}H NMR of **C1B** at room temperature in DMSO-d6

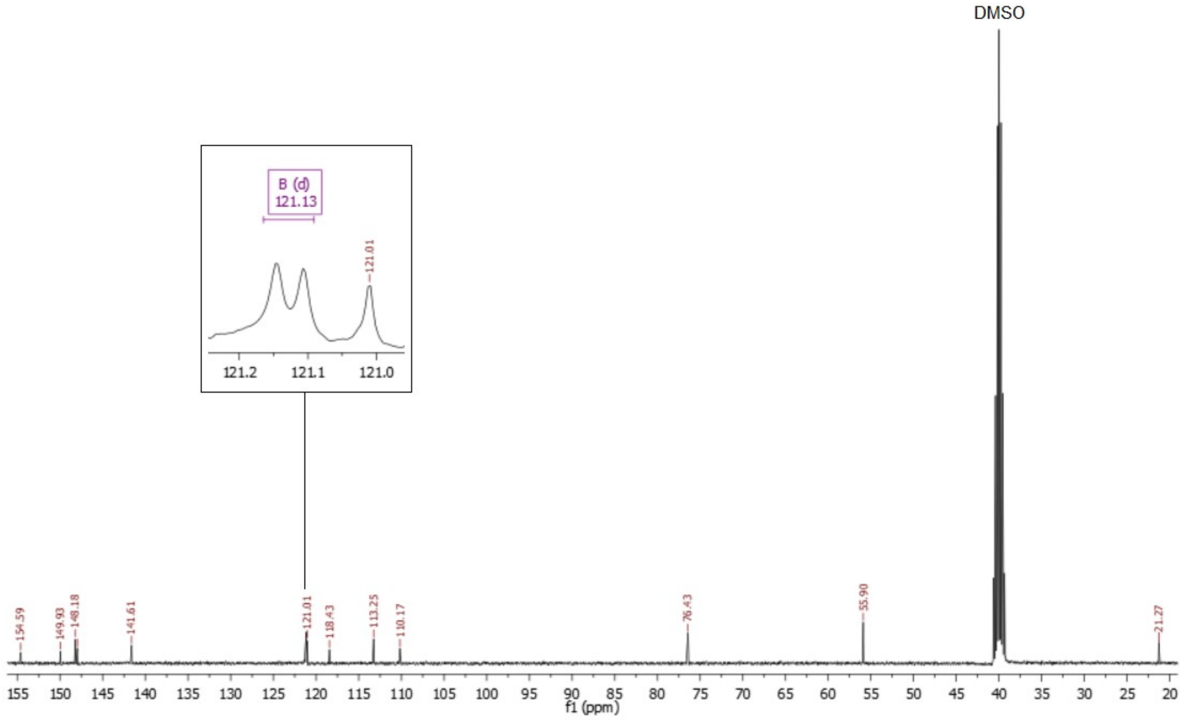

Figure 12S. $^{13}C\{^1H\}$ NMR of **C1B** at room temperature in DMSO-d6

5. FT-IR measurements

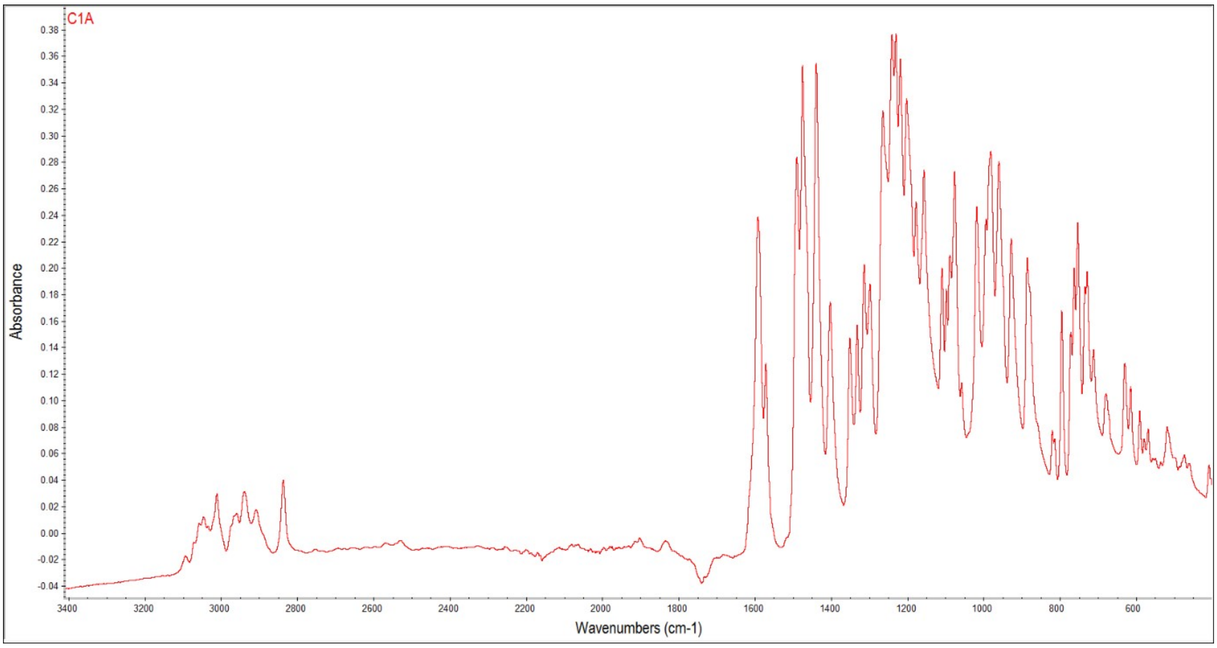

Figure 13S. FT-IR spectrum of **C1A**

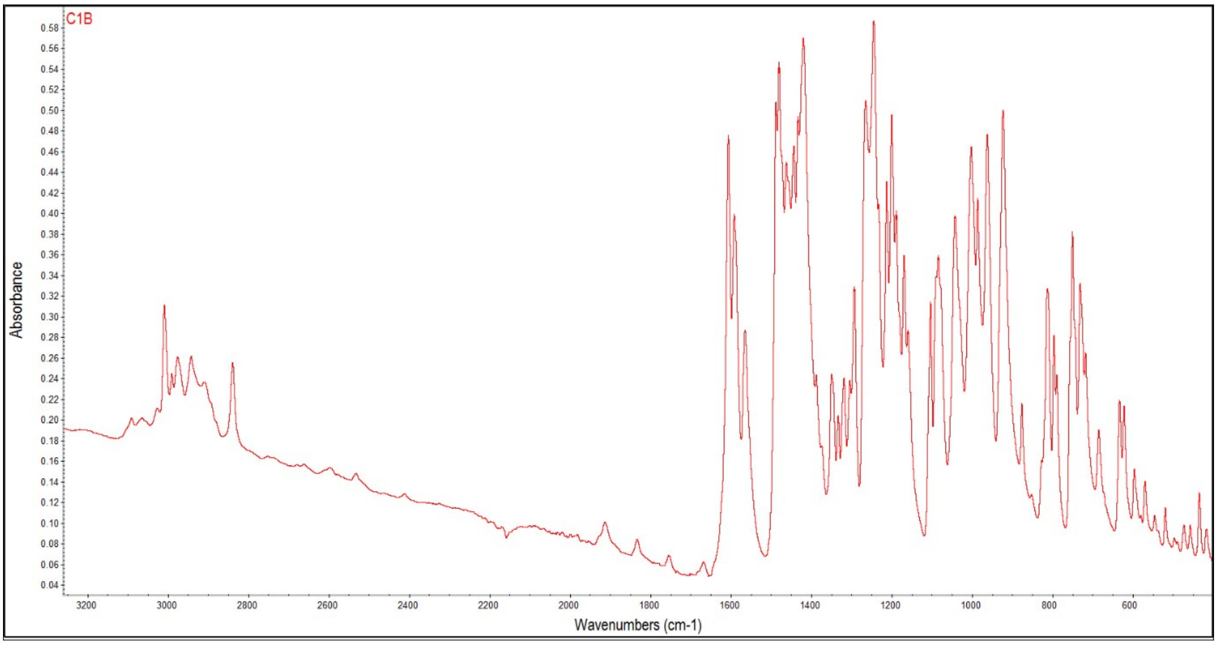

Figure 14S. FT-IR spectrum of **C1B**

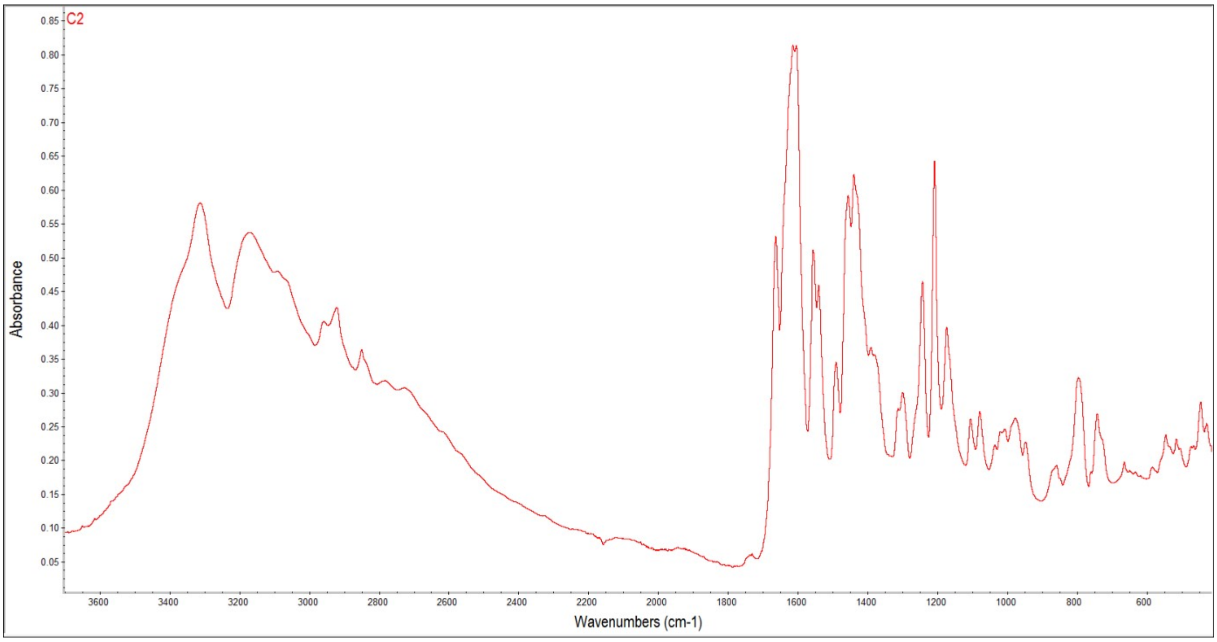

Figure 15S. FT-IR spectrum of **C2**

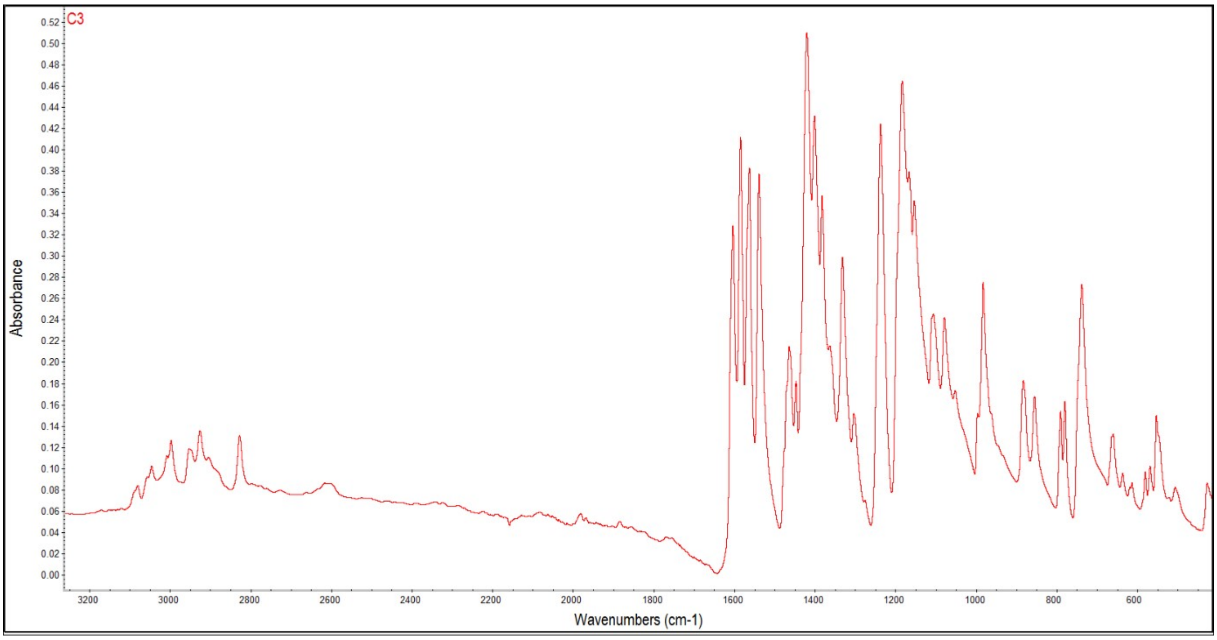

Figure 16S. FT-IR spectrum of **C3**

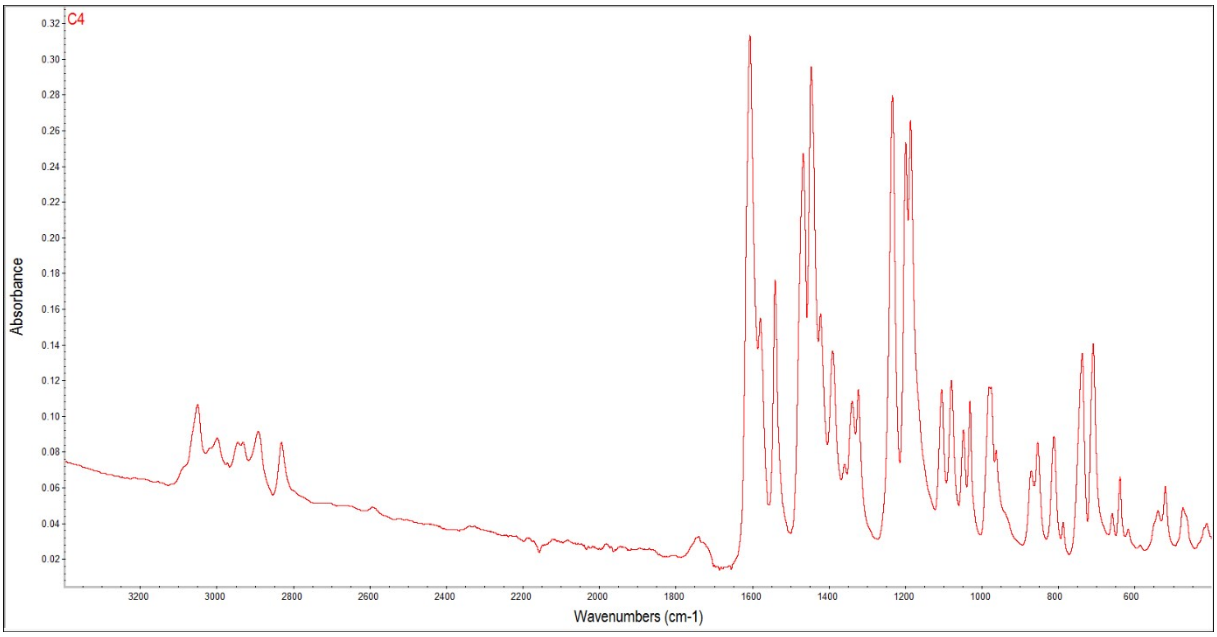

Figure 17S. FT-IR spectrum of **C4**

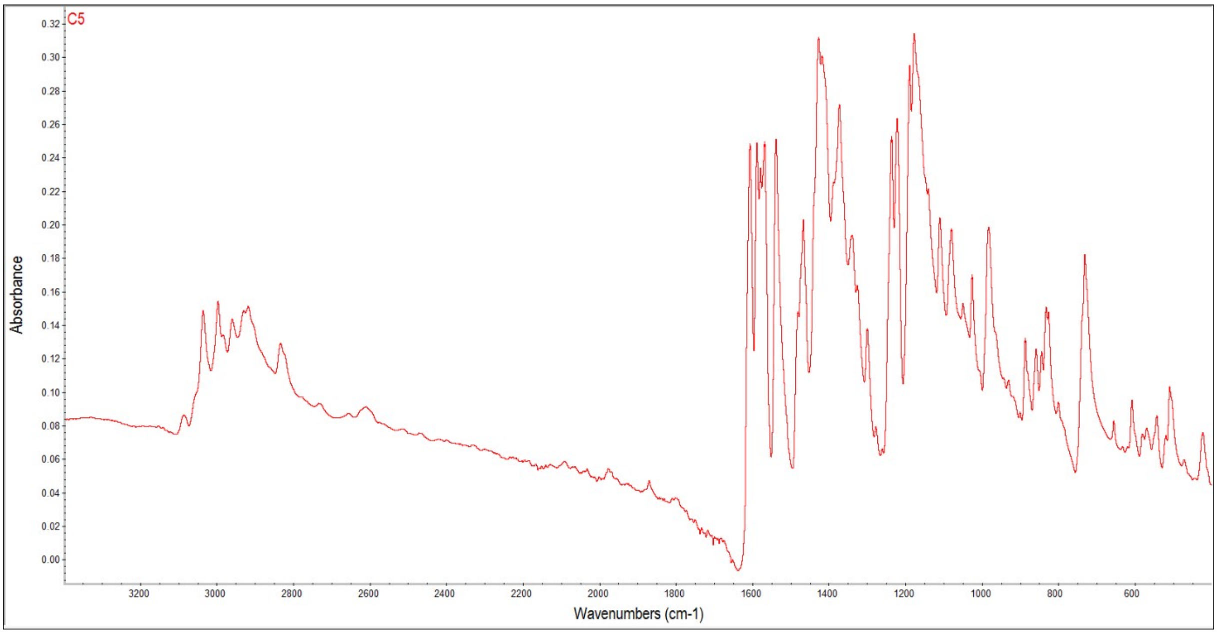

Figure 18S. FT-IR spectrum of **C5**

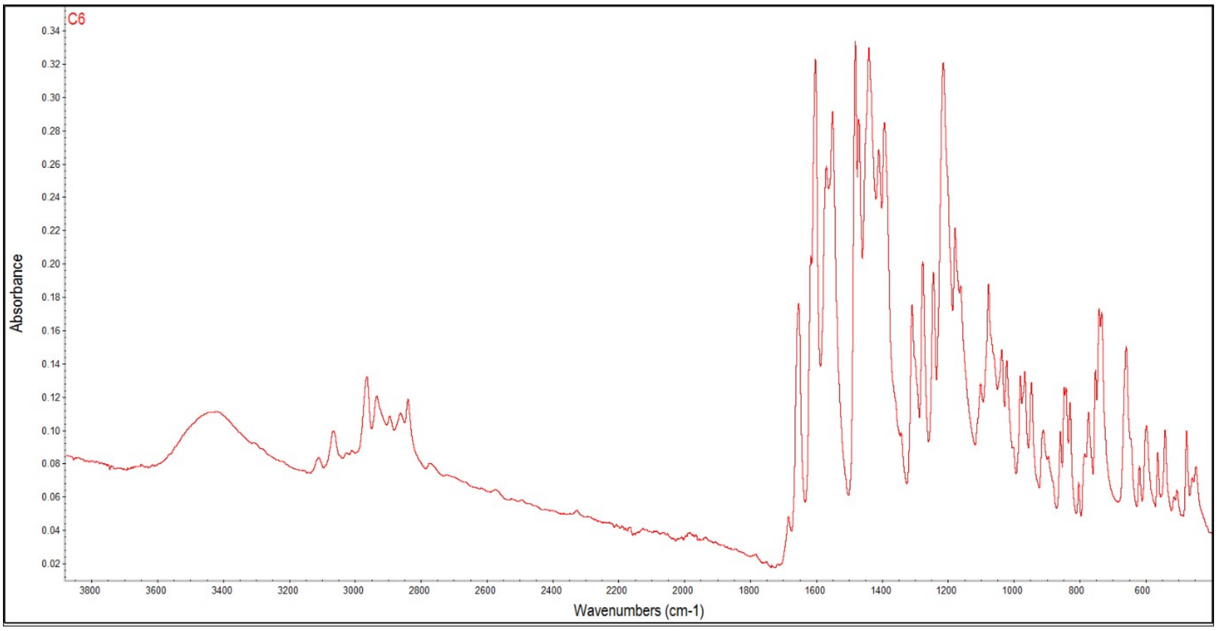

Figure 19S. FT-IR spectrum of **C6**

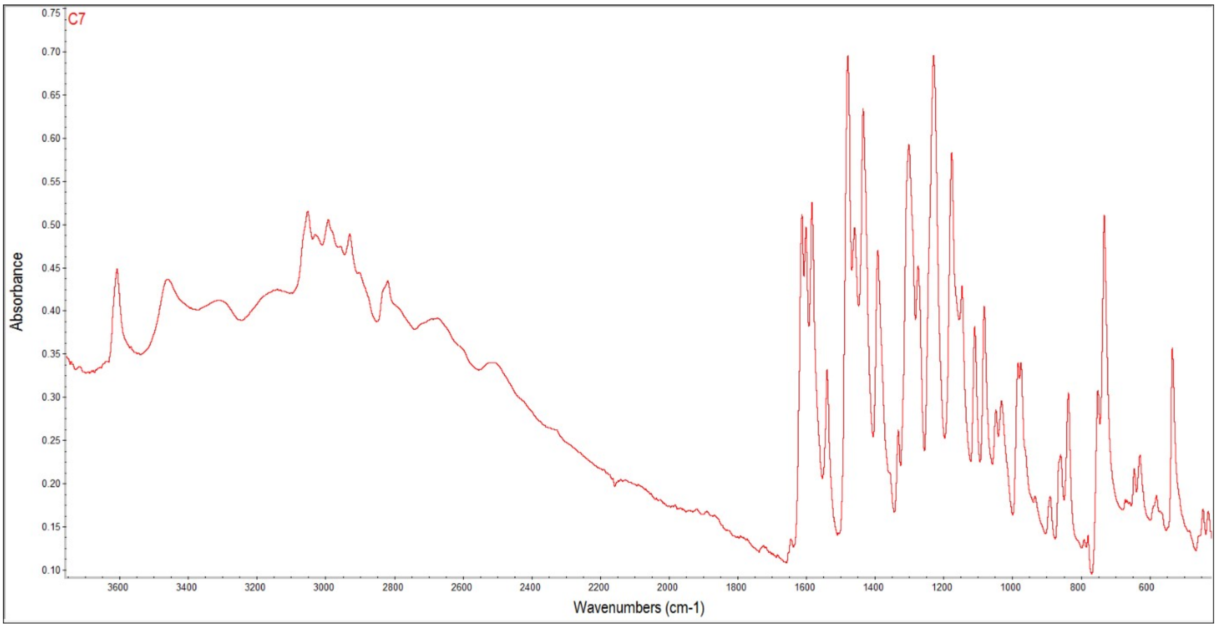

Figure 20S. FT-IR spectrum of **C7**

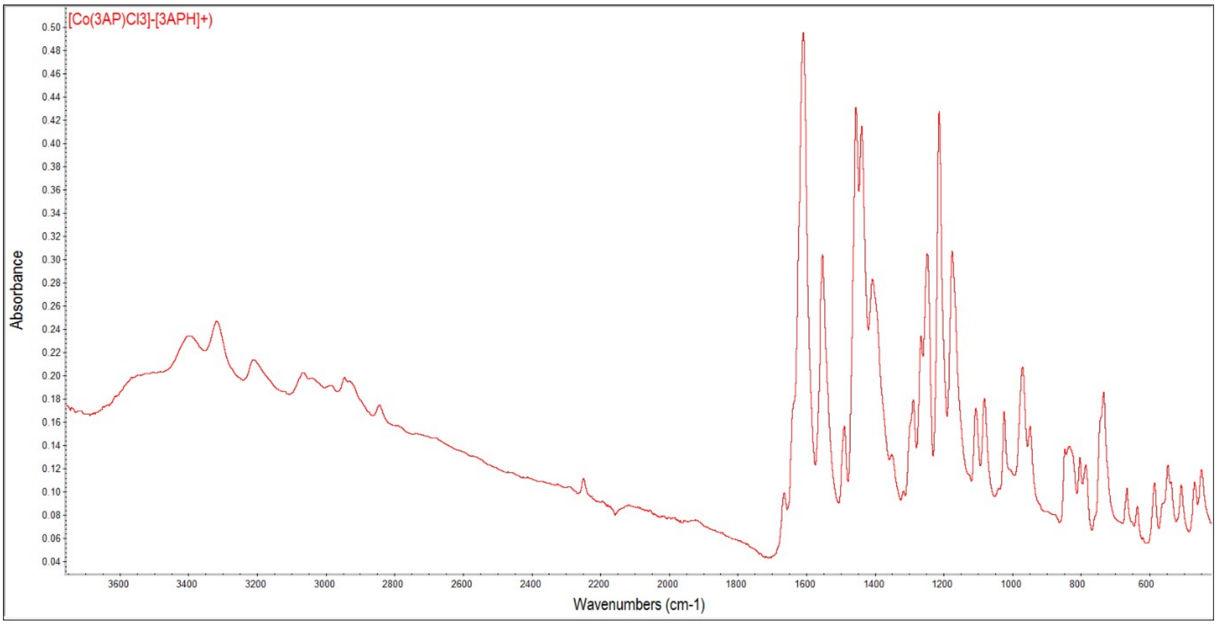

Figure 21S. FT-IR spectrum of ionic pair $[Co(3AP)Cl_3]^-[3APH]^+$)

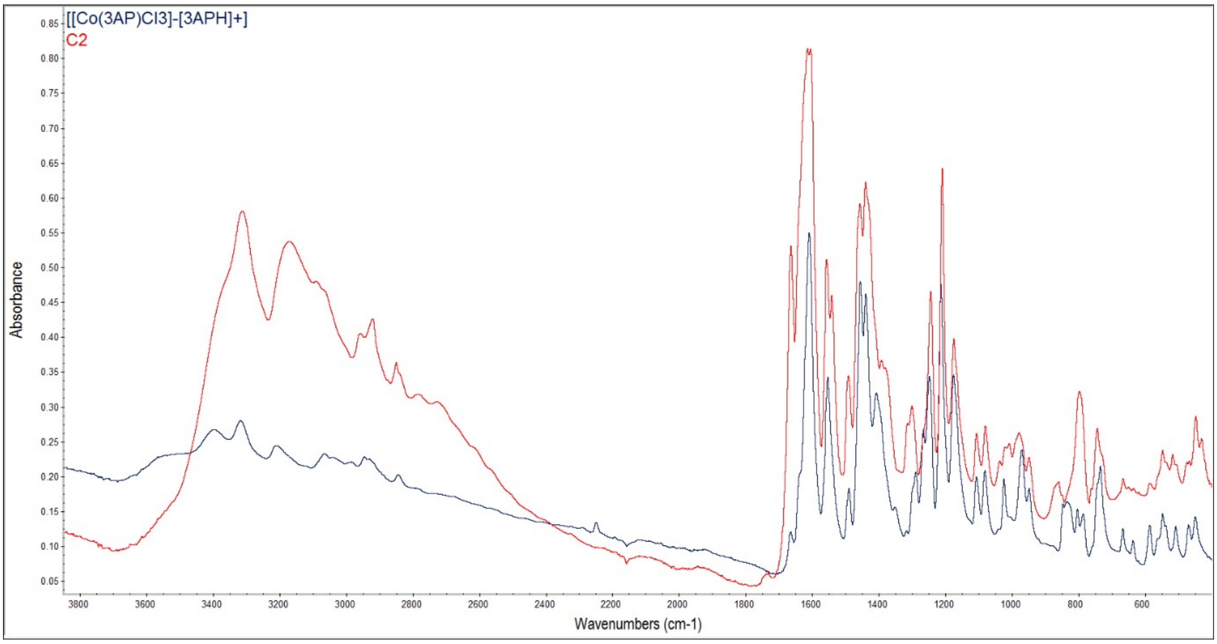

Figure 22S. FT-IR spectra of ionic complexes: $[Co(3AP)Cl_3]^-[3APH]^+$) and **C2**

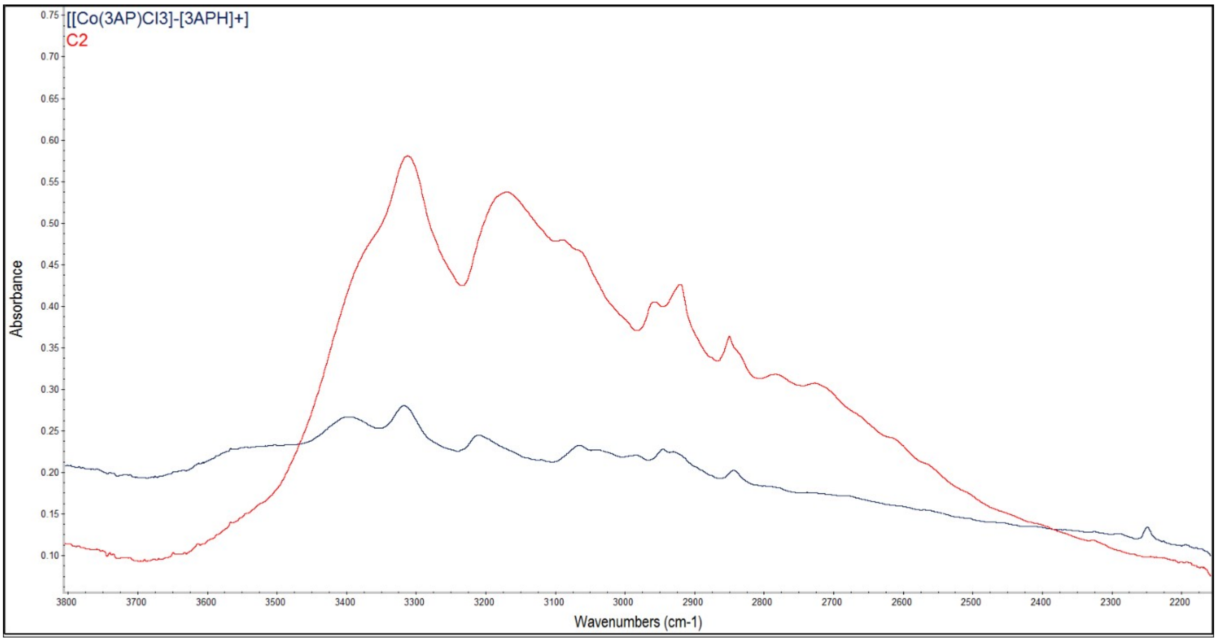

Figure 23S. FT-IR spectra of ionic complexes: $[Co(3AP)Cl_3]^-[3APH]^+$) and **C2**

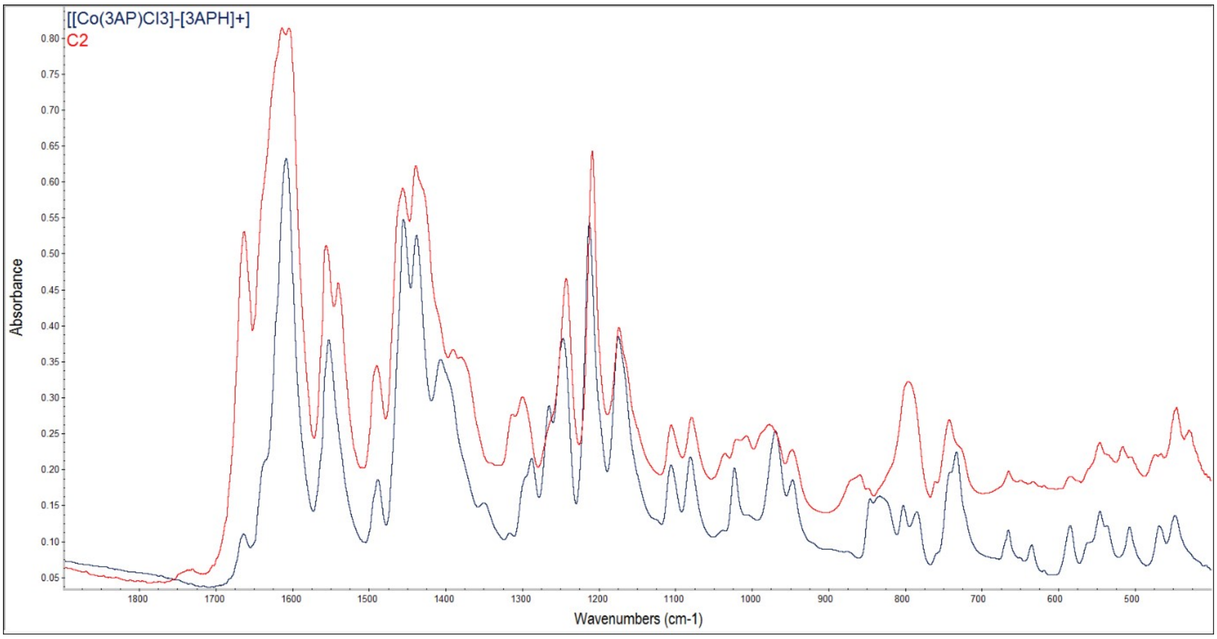

Figure 24S. Fingerprint regions of FT-IR of ionic complexes: $[Co(3AP)Cl_3]^-[3APH]^+$) and **C2.**

6. TLC of **C5**

The stability of compound **C5** in an aqueous medium was investigated. The **C5** complex was dissolved in DMSO:water mixture 1:1. After 72 hrs, the solution was analysed by TLC chromatography.

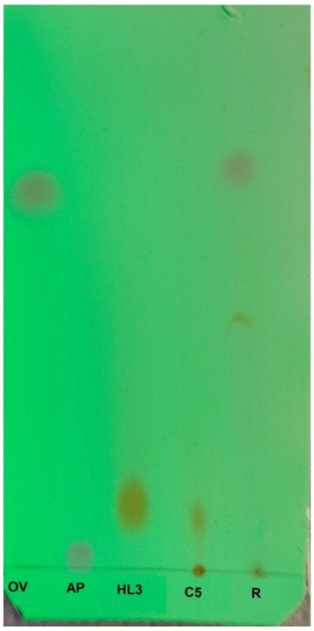

OV: o-vanillin
AP: 2-amino-5-methylpyridine
HL3: ligand
C5: complex
R: solution of **C5** in DMSO/water after 72hrs

Figure 25S. The photo of TLC plate of **C5** incubated for 72 h in DMSO/water mixture
